# Polycomb group genes are required for neuronal pruning in *Drosophila*

**DOI:** 10.1186/s12915-023-01534-0

**Published:** 2023-02-15

**Authors:** Shufeng Bu, Samuel Song Yuan Lau, Wei Lin Yong, Heng Zhang, Sasinthiran Thiagarajan, Arash Bashirullah, Fengwei Yu

**Affiliations:** 1 Temasek Life Sciences Laboratory, 1 Research Link, National University of Singapore, Singapore, 117604 Singapore; 2 Department of Biological Sciences, National University of Singapore, Singapore, 117543 Singapore; 3 Division of Pharmaceutical Sciences, University of Wisconsin-Madison, Madison, WI 53705-2222 USA

**Keywords:** Polycomb group genes, Hox genes, Dendrite pruning, Axon pruning, Ecdysone signalling, Metamorphosis, *Drosophila*

## Abstract

**Background:**

Pruning that selectively eliminates unnecessary or incorrect neurites is required for proper wiring of the mature nervous system. During *Drosophila* metamorphosis, dendritic arbourization sensory neurons (ddaCs) and mushroom body (MB) γ neurons can selectively prune their larval dendrites and/or axons in response to the steroid hormone ecdysone. An ecdysone-induced transcriptional cascade plays a key role in initiating neuronal pruning. However, how downstream components of ecdysone signalling are induced remains not entirely understood.

**Results:**

Here, we identify that Scm, a component of Polycomb group (PcG) complexes, is required for dendrite pruning of ddaC neurons. We show that two PcG complexes, PRC1 and PRC2, are important for dendrite pruning. Interestingly, depletion of PRC1 strongly enhances ectopic expression of Abdominal B (Abd-B) and Sex combs reduced, whereas loss of PRC2 causes mild upregulation of Ultrabithorax and Abdominal A in ddaC neurons. Among these Hox genes, overexpression of Abd-B causes the most severe pruning defects, suggesting its dominant effect. Knockdown of the core PRC1 component Polyhomeotic (Ph) or Abd-B overexpression selectively downregulates Mical expression, thereby inhibiting ecdysone signalling. Finally, Ph is also required for axon pruning and Abd-B silencing in MB γ neurons, indicating a conserved function of PRC1 in two types of pruning.

**Conclusions:**

This study demonstrates important roles of PcG and Hox genes in regulating ecdysone signalling and neuronal pruning in *Drosophila*. Moreover, our findings suggest a non-canonical and PRC2-independent role of PRC1 in Hox gene silencing during neuronal pruning.

**Supplementary Information:**

The online version contains supplementary material available at 10.1186/s12915-023-01534-0.

## Background

During animal development, both progressive and regressive events are crucial for shaping functional neural circuits. Neurons initially elaborate extensive projections at early developmental stages. Subsequently, they can eliminate some unnecessary or incorrect branches to form precise connections, a regressive event known as pruning [1, 2]. Pruning is a closely regulated process that selectively removes some unwanted axonal and/or dendritic branches without causing neuronal death. Insufficient pruning or over-pruning are often associated with neurological disorders in humans, such as autism spectrum disorders and schizophrenia [3–5]. Furthermore, developmental pruning involves local disassembly of axonal or dendritic branches, morphologically resembling age-dependent neurodegeneration. Thus, understanding the mechanisms of developmental pruning would provide some important insights into neurological disorders in humans.

Neuronal pruning widely occurs in both vertebrates and invertebrates. In vertebrates, neuronal pruning has been observed in layer 5 neurons of the cortex [6] as well as motor neurons at the neuromuscular junctions [7]. In holometabolous insects, such as *Drosophila*, many neurons undergo pruning during early metamorphosis [8, 9]. Mushroom body (MB) γ neurons and class IV dendritic arbourization (da) neurons (C4da or ddaC neurons) are two useful models for studying the mechanisms of developmental pruning in fly [10, 11]. MB γ neurons selectively remove their dorsal and medial axon projections via local degeneration and glia-mediated phagocytosis [12–15]. By contrast, ddaC neurons eliminate all their larval dendritic arbours without affecting their axons [16, 17]. ddaC dendrite pruning is initiated by severing of proximal dendrites, followed by dendritic fragmentation and epidermis-dependent debris clearance [16–18].

In both ddaC neurons and MB γ neurons, neuronal pruning is triggered by an ecdysone-induced multi-layer signalling cascade. First, in response to a late larval pulse of ecdysone, ecdysone receptor B1 (EcR-B1), a neuronal isoform of EcR, is upregulated and activated from the wandering third instar larval (wL3) stage onwards [12, 16]. In MB γ neurons, the expression of EcR-B1 is controlled by TGF-β signalling [19, 20], the Ftz-F1/Hr39 nuclear receptors [21], the cohesion complex [22], the BTB-zinc finger transcription factor Chinmo [23, 24], microRNA-34 [25] and the epigenetic reader Kismet [26]. Second, EcR-B1, together with its co-receptor Ultraspiracle (Usp), induces their downstream effectors to promote neuronal pruning [12, 16, 17]. In ddaC neurons, EcR-B1 and Usp induce the expression of their downstream transcription factor Sox14 [27] and the cytosolic protein Headcase [28]. The expression of Sox14 also requires a cooperation between the chromatin remodelling factor Brahma (Brm) and the histone acetyltransferase CREB-binding protein (CBP), which leads to local histone acetylation at the *sox14* locus [29]. Third, Sox14 in turn induces the expression of the F-actin disassembly factor Mical [27, 30], the Cullin1-based E3 ubiquitin ligase complex [31] and activation of the metabolic regulator AMP-activated protein kinase [32, 33] and the Nrf2-Keap1 pathway [34] to promote neuronal pruning. Although some key components of the ecdysone-induced signalling cascade have been identified, the transcriptional regulation machinery of neuronal pruning remains incomplete.

From a forward genetic screen, we isolated Sex comb on midleg (Scm), a Polycomb group (PcG) protein, which is required for dendrite pruning in ddaC neurons. PcG proteins are evolutionarily conserved epigenetic repressors that silence gene expression during development [35, 36]. PcG proteins are divided into multiple protein complexes, including Polycomb repressive complex 1 (PRC1), Polycomb repressive complex 2 (PRC2), Polycomb repressive deubiquitinase (PR-DUB), dRing-associated factors (dRAF) and Pho repressive complex (PhoRC) [35, 36]. Scm was initially identified as a potential component of PRC1 which consists of the following core components, namely Polyhomeotic (Ph), Polycomb (Pc), Posterior Sex Combs (Psc), Suppressor of zeste 2 [Su(z)2] and Sex combs extra (Sce/dRING) [37–39]. The PRC2 complex is composed of the methyltransferase Enhancer of zeste [E(z)], Extra sex combs (Esc), Suppressor of zeste 12 [Su(z)12] and Chromatin assembly factor 1, p55 subunit (Caf1-55) [35, 36]. While PRC1 is responsible for chromatin remodelling and histone ubiquitination, PRC2 mediates trimethylation of histone H3 lysine 27 (H3K27me3), a repressive marker, at the Polycomb response elements (PREs) to silence PcG target genes [40]. The best characterized PcG targets include homeobox (Hox) genes and segmentation genes such as *engrailed*, which are essential for establishment of body pattern during early embryonic development in *Drosophila* [35]. Two PRC2 components Esc and E(z) have also been reported to suppresses the Bithorax Complex (BX-C) Hox genes, such as Ultrabithorax (Ubx) and Abdominal A (Abd-A), and maintain dendritic arbours of ddaC neurons during the larval stage [41]. In fly brains, loss of the core PRC1 component Ph leads to transformation of neuronal types during metamorphosis, while loss of Pc or E(z) causes different neuronal developmental defects in MB neurons, for example, exuberant dendrites [42]. These findings highlight differential roles of PcG components in neuronal development. However, a potential role of PcG proteins in neuronal pruning, a regressive process, has not been studied. Here, we report a requirement of the PcG protein Scm for regulating dendrite pruning of ddaC neurons. We further show that PRC1 and PRC2 components are important for both dendrite pruning of ddaC neurons and axon pruning of MB γ neurons. Interestingly, our study suggests a non-canonical role of PRC1 in repressing Hox genes and promoting both types of neuronal pruning.

## Results

### Scm is cell-autonomously required for dendrite pruning in sensory neurons

**Fig. 1:**
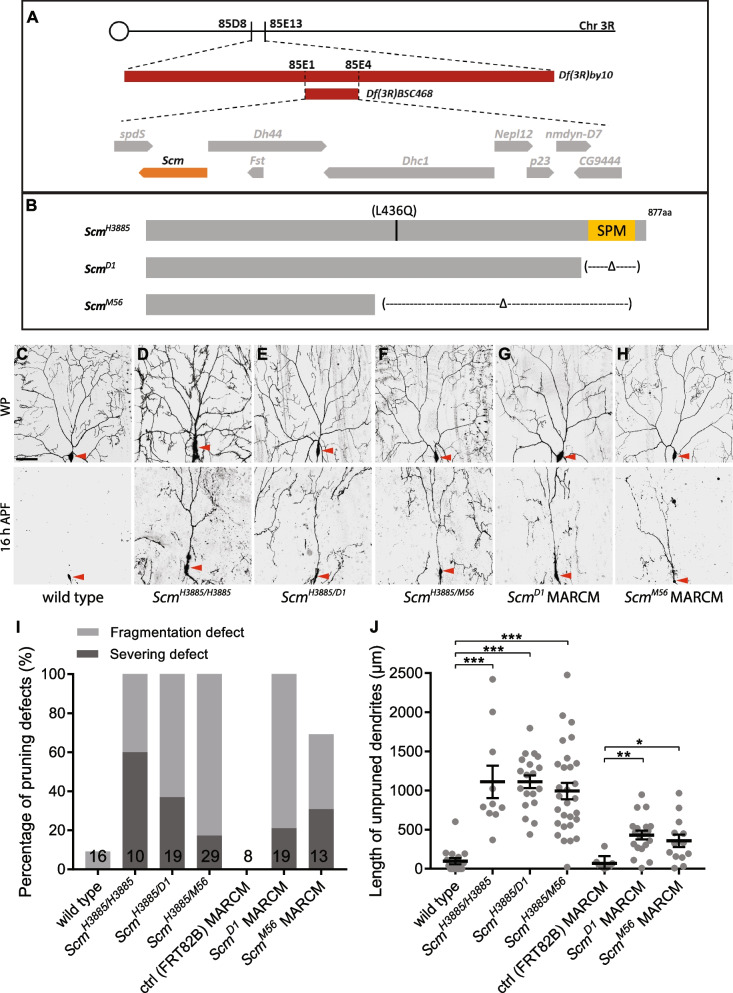
*Scm* is cell-autonomously required for dendrite pruning in ddaC neurons. **A** A schematic diagram of *Scm* gene locus and genomic mapping. **B** A schematic representation of the lesions of *Scm* mutants. **C–H** Live confocal imaging of ddaC neurons labelled by mCD8GFP at WP and 16 h APF. Somas of ddaC neurons are marked with red arrowheads. Dendrites of control ddaC neurons were pruned away at 16 h APF (**C**), whereas *Scm*^*H3885*^ homozygous mutant ddaC neurons (**D**), *Scm*^*H3885/D1*^ trans-heterozygous mutant ddaC neurons (**E**), *Scm*^*H3885/M56*^ trans-heterozygous mutant ddaC neurons (**F**), *Scm*^*D1*^ ddaC MARCM clones (**G**) or *Scm*^*M56*^ ddaC MARCM clones (**H**) exhibited the dendrite pruning defects. **I** Quantification of severing and fragmentation defects of ddaC neurons at 16 h APF. **J** Quantification of length of unpruned ddaC dendrites at 16 h APF. The number of neurons (*n*) in each group is shown on the bars. Error bars in all experiments represent ± SEM. One-way ANOVA with Bonferroni test was applied to determine significance for multiple-group comparison. *p < 0.05, **p < 0.01, ***p < 0.001. Three independent replicates were conducted. Scale bar represents 50 µm

We previously conducted a genetic screen of pupal-lethal mutations on chromosome 3R to identify new regulators of dendrite pruning in ddaC neurons [27]. In this screen, we isolated a mutant line, *l(3)H3885*, in which 60% of the homozygous mutant neurons exhibited dendrite severing defects and the rest showed dendrite fragmentation defects at 16 h after puparium formation (APF) (Fig. 1D, I, J). In contrast, wild-type ddaC neurons pruned away all their larval dendrites at this time point (Fig. 1C, I, J). *l(3)H3885* failed to complement with two deficiency lines, *Df(3R)by10* and *Df(3R)BSC468*, and was therefore mapped to the cytologic location 85E1-E4 (Fig. 1A). The subsequent complementation tests revealed that *l(3)H3885* failed to complement with two known alleles of *Scm*, *Scm*^*D1*^ and *Scm*^*M56*^ (Fig. 1B) [43, 44], indicating that the mutation locates within the *Scm* gene locus. Scm, a PcG protein, is a SPM (Scm, Ph and MBT)/SAM (sterile α motif)-motif-containing protein which was considered as a component of PRC1 and is essential for PcG-dependent gene silencing [38, 45]. Further DNA sequencing identified a point mutation, L436Q, in the coding region of *Scm* (Fig. 1B). Thus, we named this allele *Scm*^*H3885*^ hereafter. Notably, the trans-heterozygous mutant animals between *Scm*^*H3885*^ and either of two amorphic alleles, *Scm*^*D1*^ or *Scm*^*M56*^, survived to the pupal stage, whereas *Scm*^*D1*^ and *Scm*^*M56*^ homozygotes or trans-heterozygotes survived to the first or second instar larval stages (Additional file 1: Fig. S1A). These data suggest that *Scm*^*H3885*^ is likely a hypomorphic allele.

To exclude the possibility that the dendrite pruning defects associated with *Scm*^*H3885*^ is caused by other background mutations, we examined dendrite pruning defects in *Scm*^*H3885/D1*^ and *Scm*^*H3885/M56*^ trans-heterozygous mutants at 16 h APF. Consistently, we observed similar dendrite pruning defects in *Scm*^*H3885/D1*^ or *Scm*^*H3885/M56*^ mutant ddaC neurons (Fig. 1E, F, I, J). Moreover, when we overexpressed full-length Scm in *Scm*^*H3885*^ homozygous mutant ddaC neurons, the dendrite pruning defects were largely rescued (Additional file 1: Fig. S1B). Next, utilizing mosaic analysis with a repressive cell marker (MARCM) [46], we generated *Scm*^*D1*^ or *Scm*^*M56*^ homozygous mutant ddaC clones, both of which exhibited significant dendrite pruning defects (Fig. 1G–J). Finally, knocking down *Scm*, via three independent RNA interference (RNAi) lines (#1, #2, #3), also resulted in consistent dendrite pruning defects (Additional file 1: Fig. S1C). Scm protein was expressed in wild-type ddaC neurons at wL3 stage and eliminated in *Scm* RNAi #1 neurons (Additional file 1: Fig. S1D). Thus, the RNAi line #1 (#55,278) was used in the following studies. Taken together, we demonstrate that *Scm* is cell-autonomously required for dendrite pruning of ddaC neurons during early metamorphosis.

To assess if Scm also plays a role during dendritogenesis of ddaC neurons, we imaged the whole dendritic arbours of *Scm*^*D1*^ MARCM ddaC clones at 96 h after egg laying (AEL). The number of dendrite termini was reduced in *Scm*^*D1*^ mutant clones, compared to the wild-type clones (Additional file 2: Fig. S2A). Sholl analysis also indicates that the dendrite arbours of *Scm*^*D1*^ mutant ddaC neurons were simpler than the wild-type controls (Additional file 2: Fig. S2A). Therefore, Scm is required for dendrite arbourization of ddaC neurons at larval stages.

During metamorphosis, class I da neurons (ddaD/E) prune away their dendrites, whereas class III da neurons (ddaA/F) are apoptotic [17, 47]. To assess whether Scm is also required for remodelling of class I and III da neurons, we generated ddaD/E MARCM clones of *Scm*^*D1*^ mutants. While the dendrites of wild-type ddaD/E neurons were pruned at 20 h APF, *Scm*^*D1*^ mutant ddaD/E neurons still retained their major dendrites (Additional file 2: Fig. S2B). However, ddaF clones derived from *Scm*^*D1*^ mutant neurons disappeared at 16 h APF, similar to the wild-type ddaF clones (Additional file 2: Fig. S2C). Thus, Scm is required for dendrite pruning of class I da neurons, but dispensable for death of class III da neurons.

### Both PRC1 and PRC2 complexes are important for dendrite pruning in ddaC neurons

**Fig. 2:**
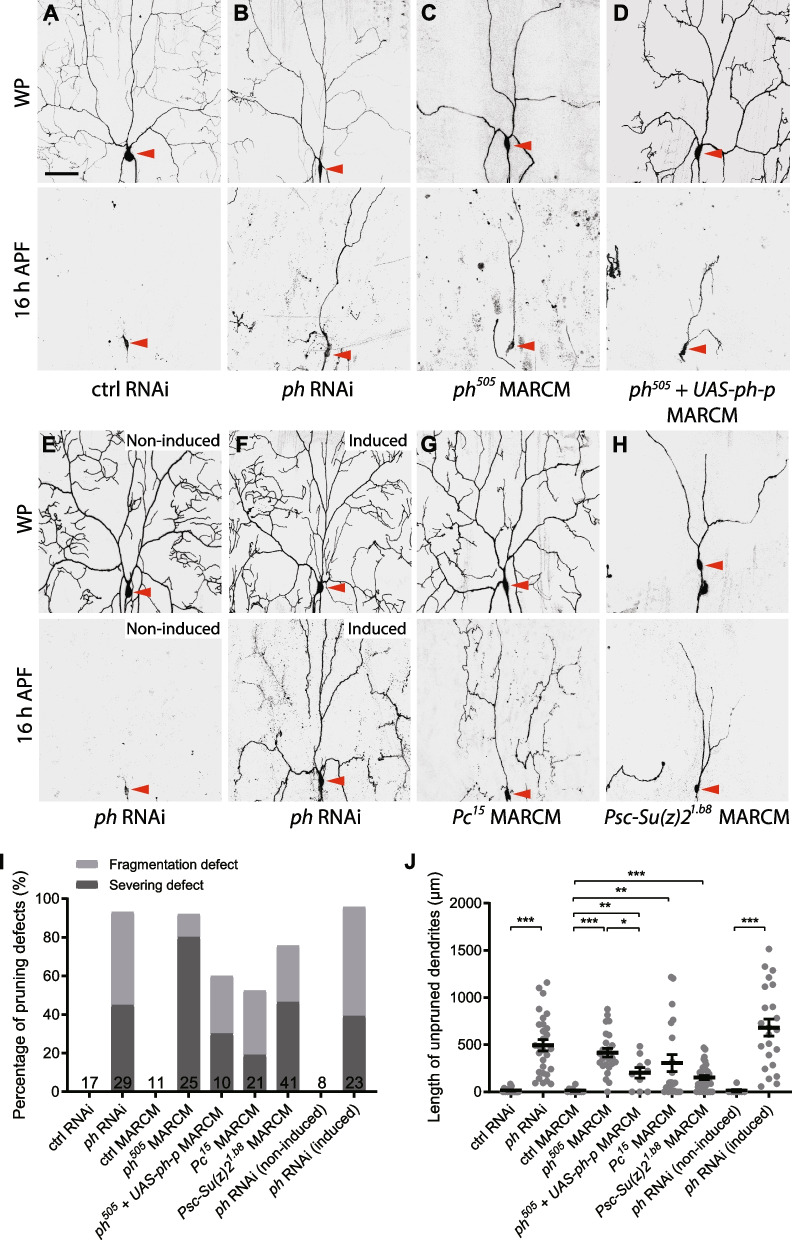
The components of PRC1 complex are required for dendrite pruning in ddaC neurons. **A–H** Live confocal images of ddaC neuron labelled by mCD8GFP at WP and 16 h APF. Somas of ddaC are marked by red arrowheads. Dendrites of control ddaC neurons were pruned away at 16 h APF (**A**, **E**), whereas *ph* RNAi*-*expressing ddaC neurons (**B**), *ph*^*505*^ ddaC MARCM clones (**C**), RU486-induced *ph* RNAi ddaC neurons (**F**), *Pc*^*15*^ ddaC MARCM clones (**G**) and *Psc-Su(z)2*^*1.b8*^ MARCM clones (**H**) exhibited the dendrite pruning defects. Overexpression of *ph-p* partially rescued the pruning defects in *ph*^*505*^ ddaC MARCM clones (**D**). **I** Quantification of severing and fragmentation defects of ddaC neurons at 16 h APF. **J** Quantification of length of unpruned ddaC dendrites at 16 h APF. The number of neurons (*n*) in each group is shown on the bars. Error bars in all experiments represent ± SEM. Two-tailed Student’s *t* test was used to determine statistical significance for pairwise comparison, whereas one-way ANOVA with Bonferroni test was applied to determine significance for multiple-group comparison. **p < 0.01, ***p < 0.001. Three independent replicates were conducted. Scale bar represents 50 µm

To investigate whether other PcG genes are also involved in regulating dendrite pruning of ddaC neurons, we systematically interrogated the potential roles of PRC1 genes using RNAi analyses (Additional file 3: Fig. S3A). Ph, a core component of PRC1 and a direct interactor of Scm [38], possesses two paralogs in *Drosophila*, namely Ph proximal (Ph-p) and Ph distal (Ph-d) [35, 48]. Multiple RNAi lines targeting either *ph-p* and/or *ph-d* exhibited notable dendrite pruning defects (Fig. 2B, I, J, Additional file 3: Fig. S3A-B; control RNAi, Fig. 2A), suggesting a requirement of these two genes. The *ph* RNAi line v50028, which targets both *ph-p* and *ph-d*, caused the most severe dendrite pruning (Additional file 3: Fig. S3A) and was thus used for the following analysis. Next, we utilized the double mutant allele *ph*^*505*^, which deletes both *ph-p* and *ph-d* [48, 49], to generate MARCM ddaC clones. Compared to *ph* RNAi knockdown, *ph*^*505*^ mutant ddaC neurons showed even higher penetrance of dendrite severing defects (Fig. 2C, I, J; wild-type clones, Fig. 2I, J). The *ph*^*505*^ mutant phenotype was partially rescued by overexpression of Ph-p (Fig. 2D, I, J), suggesting that both Ph-p and Ph-d are required for dendrite pruning. Notably, larval dendrite arbourization was severely impaired in *ph* mutant or RNAi ddaC neurons at wL3 and white prepupal (WP) stages (Fig. 2B, C, Additional file 3: Fig. S3C). The numbers of dendrite termini and branches were strongly reduced in *ph* RNAi ddaC neurons (Additional file 3: Fig. S3C). To rule out the possibility that the impaired dendrite pruning phenotype is caused by the developmental defects, we induced RNAi knockdown from the late larval stage by the GeneSwitch system. After RU486 treatment, similar dendrite pruning defects were observed at 16 h APF (Fig. 2E, F, I, J). Thus, these data highlight an important role of Ph in regulating dendrite pruning of ddaC neurons. We next tested other PRC1 components, such as Pc, Psc and the Psc-related protein Su(z)2 (Additional file 3: Fig. S3A, B). RNAi lines targeting these genes exhibited mild dendrite pruning defects (Additional file 3: Fig. S3A). Psc and Su(z)2, two adjacent paralogues, encode two highly conserved protein domains [50] and have been reported to play redundant roles in embryos, imaginal discs and follicle stem cells [51–53]. We therefore analysed their mutant phenotypes using the double mutant *Psc-Su(z)2*^*1b.8*^. MARCM analysis of *Pc*^*15*^ single mutant or *Psc-Su(z)2*^*1b.8*^ double mutant showed significant dendrite pruning defects in ddaC neurons (Fig. 2G–J). Consistently, double RNAi knockdown of Psc and Su(z)2 also led to similar dendrite pruning defects (Additional file 3: Fig. S3D). Notably, *Psc-Su(z)2*^*1b.8*^ double mutant highly resembles *ph*^*505*^ mutant in terms of impaired dendrite pruning and simplified dendrite arbours (Fig. 2C, H). Therefore, we conclude that the PRC1 complex plays a crucial role in ddaC dendrite pruning.

**Fig. 3:**
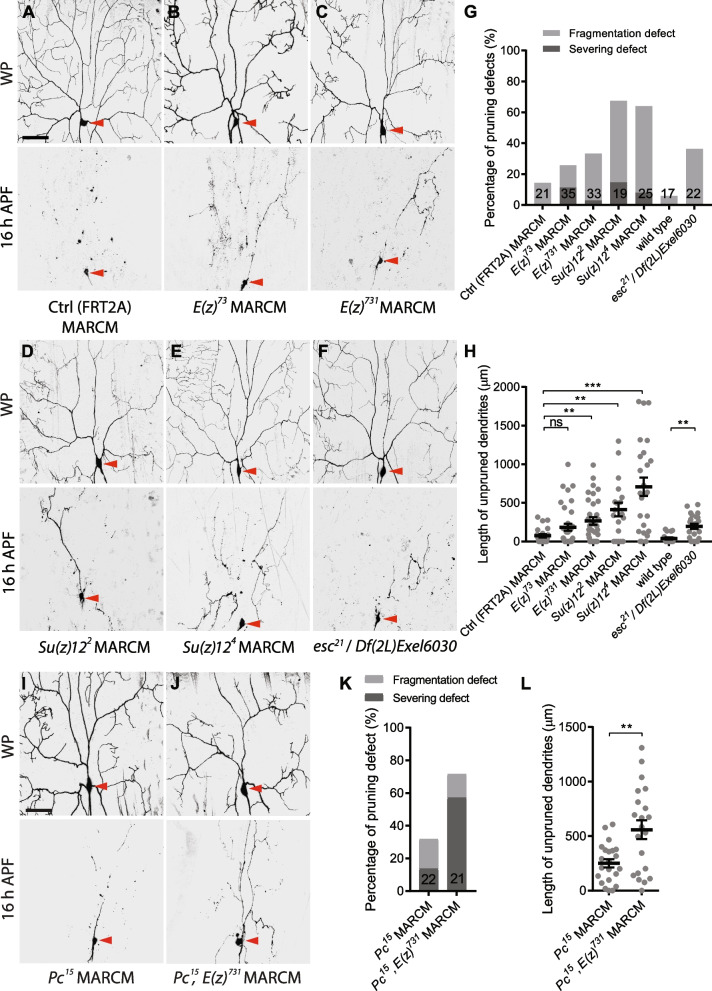
The components of PRC2 complex are important for dendrite pruning in ddaC neurons. **A–F, I,J** Live confocal images of ddaC neuron labelled by mCD8GFP at WP and 16 h APF. Somas of ddaC are marked by red arrowheads. Dendrites of control ddaC neurons were pruned away at 16 h APF (**A**), whereas *E(z)*^*73*^ ddaC MARCM clones (**B**), *E(z)*^*731*^ ddaC MARCM clones (**C**), *Su(z)12*^*2*^ ddaC MARCM clones (**D**), *Su(z)12*^*4*^ ddaC MARCM clones (**E**) and *esc*^*21*^*/Df* trans-heterozygous mutant ddaC neurons (**F**) showed mild pruning defects. Compared to *Pc*^*15*^ MARCM ddaC neurons (**I**), *Pc*^15^, *E(z)*^*731*^ double mutant MARCM clones (**J**) showed the enhanced pruning defects. **G, K** Quantification of severing and fragmentation defects of ddaC neurons at 16 h APF. **H, L** Quantification of length of unpruned ddaC dendrites at 16 h APF. The number of neurons (*n*) in each group is shown on the bars. Error bars in all experiments represent ± SEM. Two-tailed Student’s *t* test was used to determine statistical significance for pairwise comparison, whereas one-way ANOVA with Bonferroni test was applied to determine significance for multiple-group comparison. n.s., not significant, **p < 0.01, ***p < 0.001. Three independent replicates were conducted. Scale bar represents 50 µm

We next analysed whether the core components of PRC2 complex are required for dendrite pruning. Interestingly, MARCM ddaC clones derived from their loss-of-function alleles, *E(z)*^*73*^ (Fig. 3B, G, H), *E(z)*^*731*^ (Fig. 3C, G, H), *Su(z)12*^*2*^ (Fig. 3D, G, H), *Su(z)12*^*4*^ (Fig. 3E, G, H), or ddaC neurons from *esc*^*21*^*/Df(2L)Exel6030* trans-heterozygous mutant animals (Fig. 3F–H; wild-type, Fig. 3G, H) exhibited mild but significant dendrite pruning defects, as compared to the controls (Fig. 3A, G, H). Of note, the *PRC2* dendrite severing defects appeared much weaker than those in *PRC1* mutant or RNAi ddaC neurons (Figs. 2 and 3). Importantly, *E(z)*^*731*^ and *Pc*^*15*^ double mutant ddaC neurons exhibited a significant enhancement in dendrite pruning defects, as compared to *Pc*^*15*^ single mutant clones (Fig. 3I–L), suggesting their additive effect in dendrite pruning. Thus, our phenotypic analyses indicate that both PRC1 and PRC2 complexes are required for regulation of dendrite pruning of ddaC neurons.

### PRC1 and PRC2 complexes suppress distinct Hox genes in ddaC neurons

**Fig. 4:**
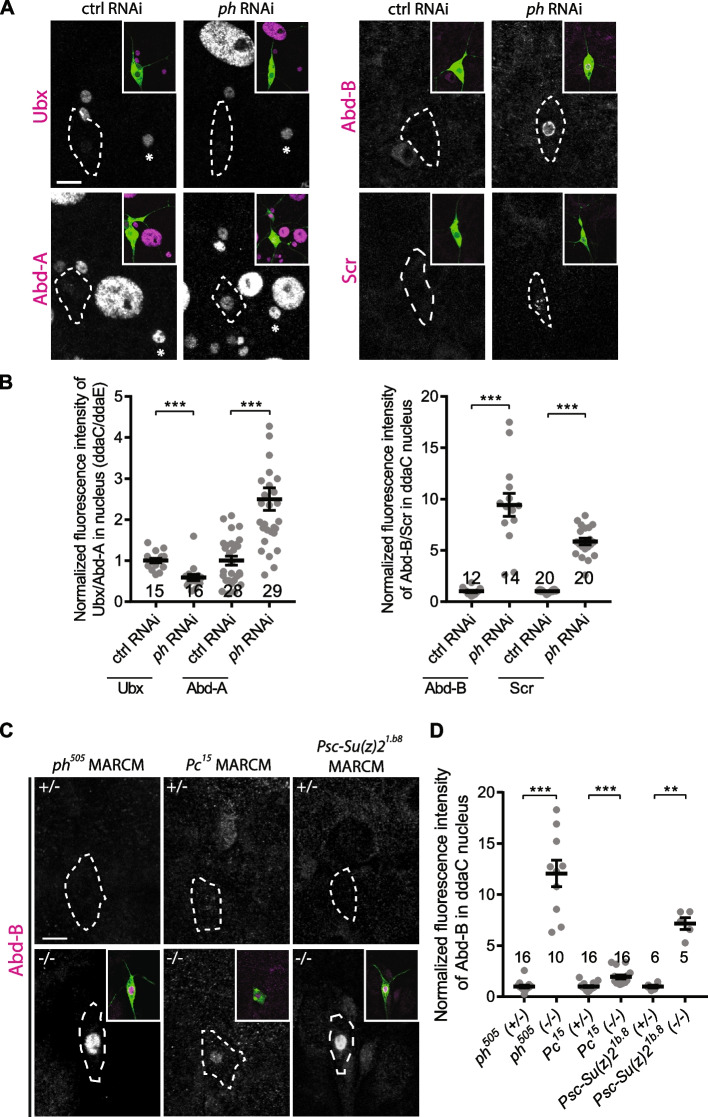
PRC1 represses Abd-B and Scr expression in ddaC neurons. **A** Confocal images of ddaC neurons (green) immuno-stained with anti-Ubx, anti-Abd-A, anti-Abd-B or anti-Scr (magenta). ddaC somas are marked by dashed lines. *ph* RNAi ddaC neurons showed de-repression of Abd-A, Abd-B and Scr, but not Ubx. **B** Quantification of Hox protein expression levels in the nuclei of ddaC neurons expressing control and *ph* RNAi. **C** MARCM ddaC clones (green) derived from *ph*^*505*^, *Pc*^*15*^ and *Psc-Su(z)2*^*1b.8*^ mutant alleles immuno-stained with anti-Abd-B (magenta). Non-clonal heterozygous ddaC controls were taken from the contralateral segment of their respective clones. ddaC somas are marked by dashed lines. **D** Quantification of Abd-B protein expression levels in the ddaC nuclei. The number of neurons (*n*) in each group is shown on the plots. Error bars in all experiments represent ± SEM. Two-tailed Student’s *t* test was used to determine statistical significance for pairwise comparison. n.s., not significant, **p < 0.01 ***p < 0.001. Three independent replicates were conducted. Scale bar represents 10 µm

PcG proteins were originally discovered as trans-acting factors to silence Hox genes during embryonic development in *Drosophila* [35, 54]. da sensory neurons are located in abdomen segments where the Bithorax complex (BX-C) Hox genes, including Ubx, Abd-A and Abd-B, are expressed [55–59]. We first attempted to check the expression of these Hox genes in ddaC neurons. Utilizing the previously published antibodies, we were able to detect endogenous expression of Ubx and Abd-A in the dorsal da neurons as well as their surrounding tissues between the abdominal segments A2-A4, while endogenous expression of Abd-B was undetectable in these segments (Additional file 4: Fig. S4A). Consistent with a previous study [41], the expression of Ubx and Abd-A in ddaC neurons was repressed at the early 3rd instar larval (eL3), wL3 and WP stages, relative to their neighbouring da neurons, such as ddaE neurons (Additional file 4: Fig. S4A). We first focused on *ph*’s function, as its RNAi knockdown showed the most severe pruning defects among PRC1 components (Fig. 2, Additional file 3: Fig. S3). Interestingly, Abd-B, which was completely silenced in wild-type da neurons, became de-silenced in *ph* RNAi ddaC neurons at wL3 (Fig. 4A, B). The expression of Abd-A but not Ubx also showed mild but significant de-repression in *ph* RNAi ddaC neurons, as compared to the control neurons (Fig. 4A, B). Moreover, among four other Hox proteins examined (Sex combs reduced/Scr, Antennapedia, Labial, Deformed), Scr, which was absent in wild-type ddaC neurons and other abdominal tissues (Fig. 4A, Additional file 4: Fig. S4A), were ectopically expressed in *ph* RNAi ddaC neurons (Fig. 4A, B). Abd-B and Scr antibodies are specific, as the proteins were largely eliminated in *ph* RNAi ddaC neurons co-expressing either *Abd-B* or *Scr* RNAi constructs (Additional file 4: Fig. S4B), respectively. Moreover, *ph*^*505*^ MARCM ddaC clones also exhibited significant increases in Abd-B and Scr protein levels at wL3 stage (Fig. 4C, D, Additional file 4: Fig. S4C). To further ascertain whether Abd-B is also derepressed in other *PRC1* mutants, we generated ddaC MARCM clones homozygous for *Pc*^*15*^ or *Psc-Su(z)2*^*1b.8*^ mutants. Importantly, ectopic expression of Abd-B was also observed in *Pc*^*15*^ or *Psc-Su(z)2*^*1b.8*^ mutant ddaC clones (Fig. 4C, D), as compared to their respective heterozygous mutant neurons located in the contralateral segments. In *Pc*^*15*^ mutant ddaC clones, however, Abd-B levels were upregulated to a lower extent than those in *ph*^*505*^ and *Psc-Su(z)2*^*1.b8*^ mutants (Fig. 4C, D). Similar to *Psc-Su(z)2*^*1b.8*^ mutant, double RNAi knockdown of Psc and Su(z)2 led to Abd-B de-repression (Additional file 4: Fig. S4D). Moreover, loss of the PRC2 component E(z) resulted in mild but significant increases in the level of Ubx and Abd-A proteins in ddaC neurons at wL3 stage (Additional file 5: Fig. S5A), consistent with the previous findings [41]. Unlike those in *ph*^*505*^ and *Psc-Su(z)2*^*1.b8*^ mutants, Abd-B and Scr expressions remained completely repressed in *E(z)*^*731*^ mutant neurons (Additional file 5: Fig. S5B). Interestingly, the expression levels of Ubx and Abd-A, but not Abd-B or Scr, were significantly increased in *Scm*^*D1*^ MARCM ddaC clones (Additional file 6: Fig. S6A-B), suggesting that *Scm* mutant resembles *PRC2* mutants, rather than *PRC1* mutants, with reference to Hox gene repression. In support of this, a recent study has reported that Scm also complexes and colocalizes with PRC2 in embryos and cultured cells [60], raising the possibility that Scm might function intimately with PRC2 proteins in ddaC neurons. Thus, PRC1 and PRC2 proteins repress distinct Hox genes in ddaC neurons during dendrite pruning.

### Overexpression of Hox genes inhibits dendrite pruning

**Fig. 5:**
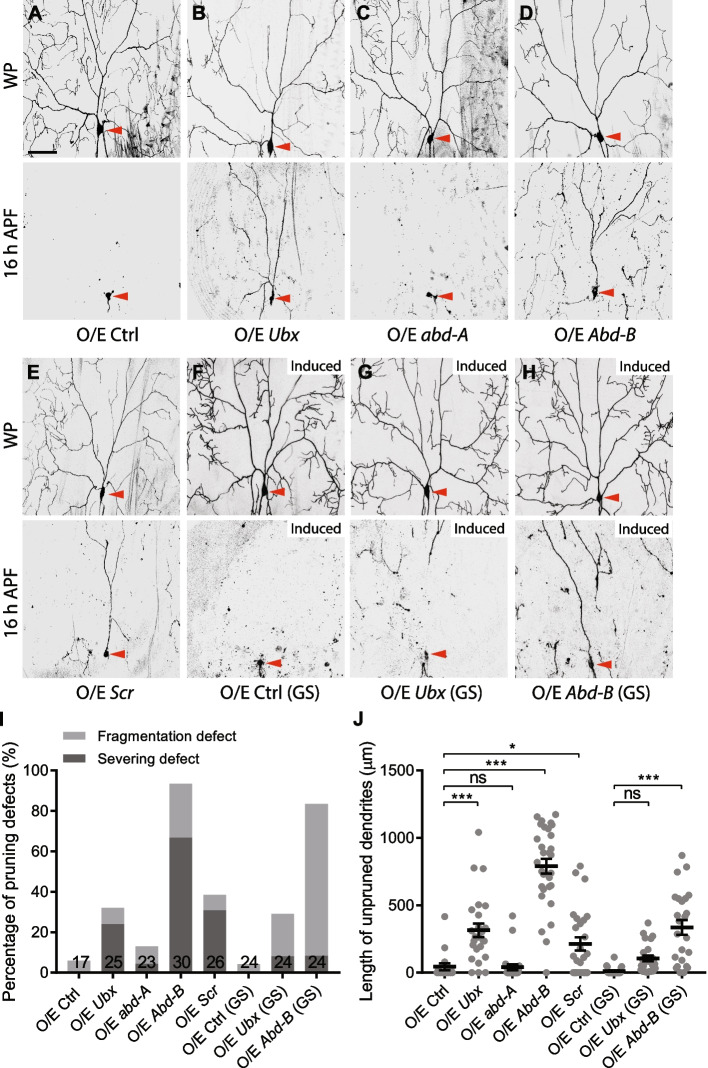
Ectopic expression of Hox genes inhibits dendrite pruning. **A–H** Live confocal images of ddaC neurons at WP and 16 h APF. Somas of ddaC are marked by red arrowheads. Overexpression of Ubx (**B**), or Abd-B (**D**), Scr (**E**), but not Abd-A (**C**) in ddaC neurons caused significant dendrite pruning defects, as compared to the control (**A**). RU486-induced late larval expression of Abd-B (**H**), but not Ubx (**G**), via the GeneSwitch system, impaired dendrite pruning in ddaC neurons, as compared to the control (**F**). **I** Quantification of severing and fragmentation defects of ddaC neurons at 16 h APF. **J** Quantification of length of unpruned ddaC dendrites at 16 h APF. The number of neurons (*n*) in each group is shown on the bars. Error bars in all experiments represent ± SEM. One-way ANOVA with Bonferroni test was applied to determine significance for multiple-group comparison. n.s., not significant, *p < 0.05, ***p < 0.001. Three independent replicates were conducted. Scale bars represent 50 µm

Next, we hypothesized that derepressed Hox genes lead to the dendrite pruning defects in *PcG* mutants. To test this possibility, we overexpressed Ubx, Abd-A, Abd-B or Scr in ddaC neurons and imaged the dendrites at 16 h APF. Interestingly, overexpression of Ubx (Fig. 5B, I, J), Abd-B (Fig. 5D, I, J) and Scr (Fig. 5E, I, J), but not Abd-A (Fig. 5C, I, J), significantly inhibited dendrite pruning, as compared to the control neurons (Fig. 5A, I, J). The quantification data indicate that Abd-B overexpression elicits the strongest effect among these four Hox genes examined (Fig. 5I, J). Moreover, dendrite arbourization was severely impaired at WP stage when Ubx or Abd-B was overexpressed in ddaC neurons (Fig. 5B, D). We therefore examined the dendrite pruning phenotype after GeneSwitch-induced Hox gene overexpression. Notably, induced expression of Abd-B at late larval stage resulted in significant dendrite pruning defects in ddaC neurons (Fig. 5H–J; control, Fig. 5F), whereas the Ubx effect was merely marginal (Fig. 5G, I, J). Taken together, these data suggest that PcG components might facilitate dendrite pruning via suppression of Hox genes.

To test whether the *ph* phenotype is caused by Abd-B or Scr upregulation, we further knocked down either of them in the *ph* RNAi ddaC neurons. The effectiveness of *Abd-B* and *Scr* RNAi lines was validated by the absence of their antibody staining in ddaC neurons (Additional file 4: Fig. S4B). Interestingly, neither Abd-B nor Scr knockdown was able to suppress the dendrite pruning defects in *ph* RNAi ddaC neurons (Additional file 7: Fig. S7A). Thus, these data suggest that in addition to Abd-B and Scr, there might exist other unknown targets that also inhibit dendrite pruning in *ph* RNAi neurons.

### Ph knockdown and Abd-B overexpression led to downregulation of Mical expression

It has been reported that loss of *ph* function leads to an alteration in neuronal identities in brains during metamorphosis [42]. To rule out the possibility that the identity of ddaC neurons is altered upon *ph* knockdown, we examined the expression of two transcription factors, Cut and Knot/Collier. While Cut is expressed at a relatively high level in C4da neurons [61], Knot/Collier is specifically present in these neurons [62–64]. We found that both Cut and Knot were still expressed in *ph* RNAi ddaC neurons (Additional file 7: Fig. S7B), although their protein levels were reduced. These data suggest that loss of Ph does not cause cell-fate transformation in ddaC neurons.

**Fig. 6:**
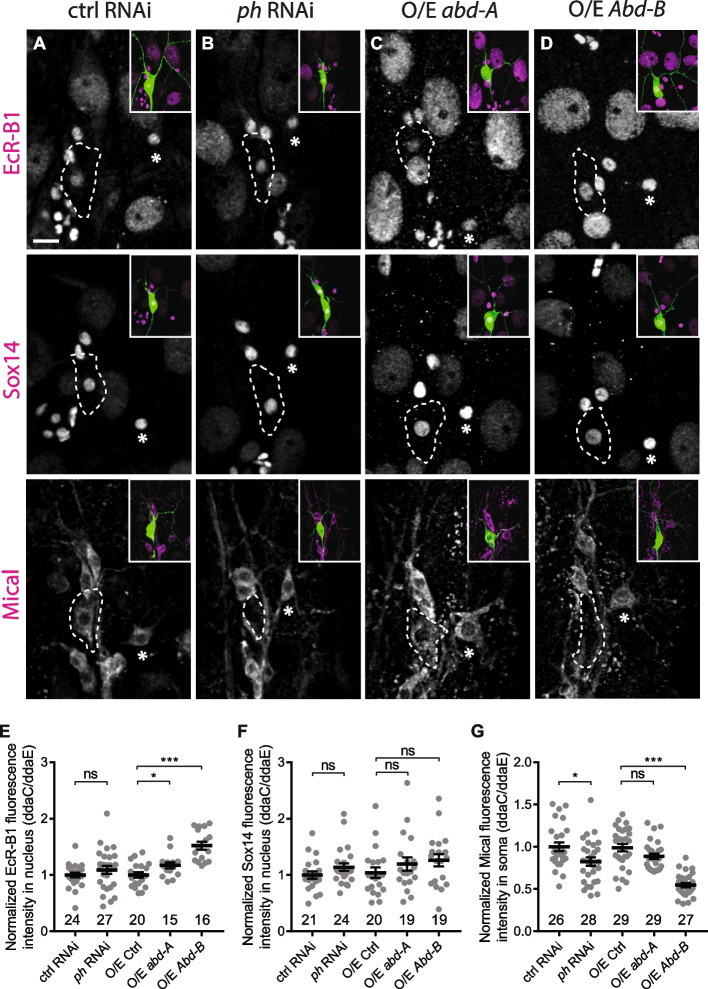
Ph knockdown and Abd-B overexpression led to downregulation of Mical expression. **A–D** Confocal images of ddaC neurons (green) at WP stage immuno-stained with anti-EcR-B1, anti-Sox14 or anti-Mical (magenta). ddaC somas are marked by dashed lines. ddaE somas are marked with asterisks. EcR-B1 and Sox14 expression levels were not reduced in *ph* RNAi (**B**), Abd-A-overexpressing (**C**) and Abd-B-overexpressing ddaC neurons (**D**). Mical expression levels were unaffected in Abd-A-overexpressing ddaC neurons (**C**) but significantly reduced in *ph* RNAi (**B**) and Abd-B-overexpressing ddaC neurons (**D**). **E–G** Quantification of EcR-B1, Sox14 and Mical expression levels. The number of neurons (*n*) in each group is shown on the plots. Error bars in all experiments represent ± SEM. Two-tailed Student’s *t* test was used to determine statistical significance for pairwise comparison, whereas one-way ANOVA with Bonferroni test was applied to determine significance for multiple-group comparison. n.s., not significant, *p < 0.05, ***p < 0.001. Three independent replicates were conducted. Scale bars represent 10 µm

It has been reported that EcR-B1, Sox14 and Mical, three components of ecdysone signalling, are upregulated in ddaC neurons to promote dendrite pruning during early metamorphosis [16, 27]. We next assessed if Ph is required for upregulation of EcR-B1, Sox14 and Mical at WP stage. EcR-B1 and Sox14 protein levels remained unaltered in *ph* RNAi neurons (Fig. 6B, E, F), as compared to the controls (Fig. 6A, E, F). By contrast, Mical protein levels were significantly reduced upon *ph* knockdown (Fig. 6B, G). Thus, Ph is required for upregulation of Mical, rather than EcR-B1 and Sox14. In addition, we also examined Mical expression in other *PcG* mutants including *E(z)*^*731*^, *Su(z)12*^*2*^, *Scm*^*D1*^ and *Pc*^*15*^ mutants as well as *Psc-Su(z)2* double RNAi ddaC neurons. Interestingly, Mical levels were downregulated at WP stage in *Pc*^*15*^ mutant and *Psc-Su(z)2* double RNAi neurons but not in *E(z)*^*731*^, *Su(z)12*^*2*^ or *Scm*^*D1*^ mutant neurons (Additional file 8: Fig. S8A), indicating that the expression of Mical depends on the core PRC1 components Ph, Pc and Psc-Su(z)2, but not on the PRC2 components E(z), Su(z)12 and Scm. Taken together, our results suggest a non-canonical and PRC2-independent role of PRC1 complex in regulating Mical expression and dendrite pruning.

We next assessed whether overexpression of Hox genes inhibits ecdysone signalling, resembling *ph* knockdown. To this end, we examined EcR-B1, Sox14 and Mical expression in Abd-A- or Abd-B-overexpressing ddaC neurons at WP stage. Overexpression of Abd-B led to a slight but significant increase in EcR-B1 expression but did not affect Sox14 upregulation (Fig. 6D–F). Interestingly, Abd-B overexpression significantly downregulated Mical expression (Fig. 6D, G). Ph and Abd-B are required for *mical* transcription, as the *mical* reporter *mical1-lacZ* was downregulated at WP stage in *ph* RNAi or Abd-B-overexpressing ddaC neurons (Additional file 8: Fig. S8B). Overexpression of Mical significantly suppressed the dendrite pruning defects in *ph* RNAi or Abd-B-overexpressing ddaC neurons (Additional file 8: Fig. S8C), suggesting that Ph and Abd-B regulate dendrite pruning at least partly through Mical transcription. By contrast, Abd-A overexpression did not inhibit EcR-B1, Sox14 or Mical upregulation (Fig. 6C, E, F, G), consistent with no effect of Abd-A overexpression on dendrite pruning (Additional file 5: Fig. 5C). These data suggest that Ph might promote Mical upregulation via Abd-B silencing. Unexpectedly, Mical expression was not restored in *ph* and *Abd-B* double RNAi ddaC neurons, as the Mical protein levels in these neurons remained absent, like the *ph* RNAi controls (Additional file 7: Fig. S7C). The failure to restore Mical expression may also explain why Abd-B knockdown alone did not rescue the dendrite pruning defects in *ph* RNAi ddaC neurons (Additional file 7: Fig. S7A). Thus, these results suggest that Ph promotes Mical upregulation and dendrite pruning by suppressing multiple targets.

### Ph is required for axonal pruning and Abd-B silencing in MB γ neurons

**Fig. 7:**
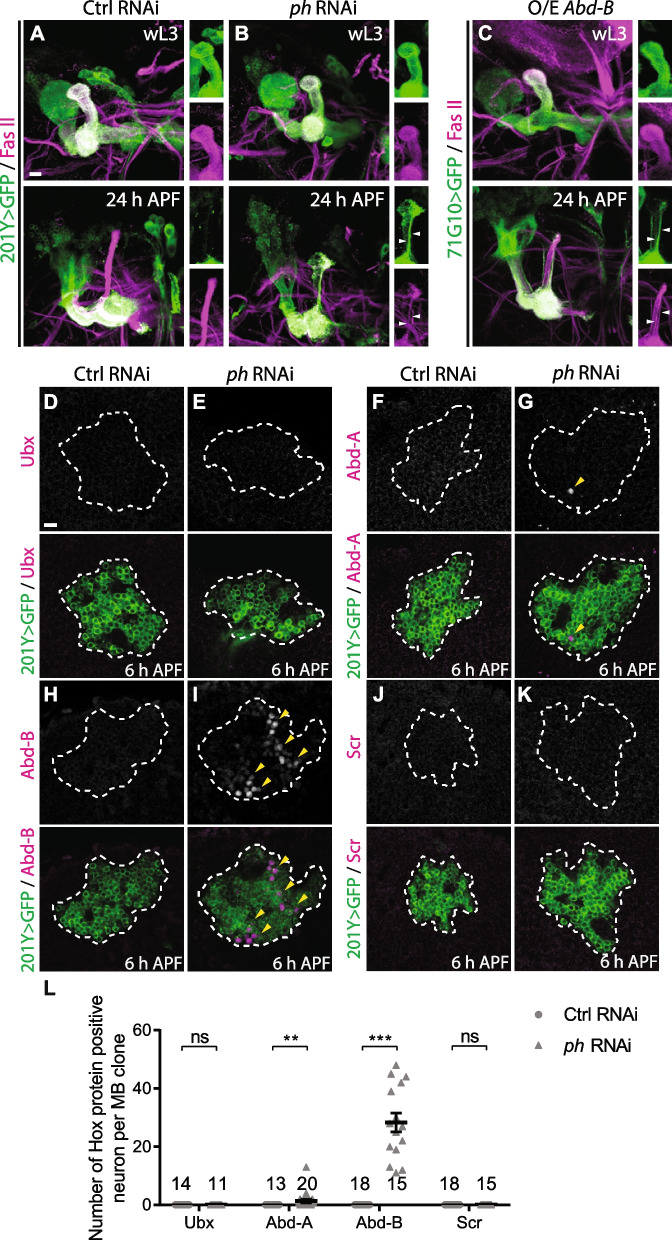
Ph is required for axonal pruning and Abd-B silencing in MB γ neurons. **A–C** Confocal images of MB γ neurons expressing mCD8GFP driven by *201Y-Gal4* and co-stained with anti-GFP (green) and anti-FasII (magenta) at wL3 stage and 24 h APF. White arrowheads point to the unpruned axons of γ neurons at 24 h APF as co-labelled by GFP and FasII. Axons of control MB γ neurons were pruned away at 24 h APF (**A**), whereas *ph* RNAi #1 (**B**) or Abd-B-overexpressing (**C**) MB γ neurons exhibited axon pruning defects. **D–K** Confocal images of MB γ neurons expressing mCD8GFP co-stained with anti-GFP (green) and anti-Ubx (**D,E**), anti-Abd-A (**F,G**), anti-Abd-B (**H,I**) or anti-Scr (**J,K**) (magenta) at 6 h APF. Somas of MB γ neurons are labelled by dashed lines. Ubx and Scr were not expressed in either control or *ph* RNAi neurons. Abd-A expression was derepressed in a few of γ neurons expressing *ph* RNAi, whereas Abd-B is derepressed in many *ph* RNAi γ neurons (indicated by arrowheads). **L** Quantification of the numbers of Ubx, Abd-A, Abd-B or Scr-positive MB γ neurons each brain lobe. The number of samples (*n*) in each group is shown on the plots. Error bars in all experiments represent ± SEM. Two-tailed Student’s *t* test was used to determine statistical significance for pairwise comparison. n.s., not significant, **p < 0.01, ***p < 0.001. Three independent replicates were conducted. Scale bars represent 10 µm

During early metamorphosis, MB γ neurons undergo axon pruning, which is also triggered by ecdysone signalling [10, 11]. We then examined a potential requirement of PcG genes for axon pruning in MB γ neurons. *201Y-Gal4* or *71G10-Gal4*-driven mCD8GFP was utilized to visualize axon branches of MB γ neurons in combination with FasII (1D4) antibody staining [22]. *201Y-Gal4* driver labels all the γ neurons as well as a small portion of late-born α/β neurons, whereas *71G10-Gal4* is specifically expressed in γ neurons [23, 65]. At 24 h APF, the control MB γ neurons selectively pruned away their axonal branches (*n* = 32; Fig. 7A, Additional file 9: Fig. S9A). RNAi knockdown of Ph (Fig. 7B) or Scm (Additional file 9: Fig. S9A) did not affect formation of their dorsal and medial branches at wL3 stage. Notably, Ph (100%, *n* = 23; Fig. 7B) or Scm knockdown (100%, *n* = 39; Additional file 9: Fig. S9A) resulted in axon pruning defects in MB γ neurons, as shown by the co-labelling of GFP and FasII on the remaining axonal branches (arrowheads in the insets of Fig. 7B, Additional file 9: Fig. S9A). Interestingly, MB α branches were lost at 24 h APF when Ph was knocked down by *201Y-Gal4* (Fig. 7B), suggesting that Ph is required for the formation of late-born α/β neurons. Thus, these findings indicate an important role of PcG genes in regulating axon pruning of MB γ neurons.

We next investigated whether Hox genes are also repressed by Ph and Scm proteins in MB γ neurons, like in ddaC neurons. As controls, Ubx, Abd-A, Abd-B and Scr proteins were absent in wild-type MB γ neurons at 6 h APF (Fig. 7D, F, H, J). Interestingly, RNAi knockdown of Ph resulted in ectopic expression of Abd-B in some but not all MB γ neurons (Fig. 7I, L; arrowheads), compared to the controls (Fig. 7H, L). On average, approximately 28 neurons were Abd-B-positive in a single section of MB neuroblast clones (Fig. 7L). Ectopic Abd-A expression was occasionally detected in a few neurons depleted of Ph (Fig. 7G, L; arrowheads). However, *Ubx* and *Scr* genes were still silenced in *ph* RNAi γ neurons at 6 h APF (Fig. 7E, K, L). We next assessed whether ectopic expression of Abd-B is sufficient to block axon pruning in MB γ neurons. Notably, under the control of *71G10-Gal4* driver, Abd-B overexpression (100%, *n* = 25; Fig. 7C) led to axon pruning defects at 24 h APF, as compared to the control overexpression (0%, *n* = 23; data not shown). Thus, like those in ddaC sensory neurons, Ph silences Abd-B and promotes axonal pruning of MB γ neurons. In contrast, RNAi knockdown of Scm did not lead to de-silencing of four Hox genes examined (Additional file 9: Fig. S9B). Taken together, our data suggest that Scm and Ph regulate axon pruning of MB γ neurons via distinct targets or mechanisms.

## Discussion

It has been well documented that PcG genes regulate various developmental processes, such as body patterning and cell fate determination [35, 66, 67]. During the development of nervous systems, PcG genes are also involved in neuronal differentiation, dendrite maintenance and neural stem cell proliferation in invertebrates and vertebrates [41, 42, 68, 69]. However, their function in neuronal remodelling remains elusive. Here, via extensive RNAi and mutant analyses, we unravel important roles of PRC1 and PRC2 components in regulating dendrite pruning of ddaC sensory neurons during *Drosophila* metamorphosis. Interestingly, silencing of the BX-C Hox gene *Abd-B* in ddaC neurons is specifically mediated by the PRC1 components, such as Ph, Pc and Psc-Su(z)2, but not by the PRC2 complex. Abd-B overexpression alone is sufficient to inhibit dendrite pruning. We further show that Ph knockdown and Abd-B overexpression inhibit ecdysone signalling by selectively downregulating Mical expression in ddaC neurons (Additional file 10: Fig. S10). Finally, the core PRC1 component Ph is also required for axon pruning and Abd-B silencing in MB γ neurons, suggesting a conserved function of the PRC1 complex in regulating two types of neuronal pruning. Thus, this study suggests that PRC1 plays a non-canonical and PRC2-independent role in Abd-B silencing and neuronal pruning.

### Scm likely acts as a component of PRC2 during dendrite pruning

Scm was isolated as an important PcG protein required for Hox gene silencing in fly embryogenesis [44, 70, 71]. Scm was originally classified as a PRC1 component because it, via its SPM/SAM domain, mediates the protein interaction with the core PRC1 component Ph in in vitro pulldown assays [38]. However, in subsequent in vivo assays, Scm was unable to co-immunoprecipitate with other PRC1 components in fly embryos [45]. Moreover, PRC1 components, including Ph, Psc/Su(z)2 and Pc, but not Scm, are sufficient to maintain chromatin structure and repress transcription in vitro [72, 73]. These findings suggest that Scm is unlikely to function as an essential component of PRC1. In this study, we identify Scm as an important regulator of neuronal pruning in both ddaC and MB γ neurons (Fig. 1, Additional file 9: Fig. S9). Multiple lines of evidence suggest that during neuronal pruning, Scm likely behaves as a PRC2 component, instead of a component of PRC1. First, *Scm*^*D1*^ mutant ddaC neurons exhibited de-repression of *Ubx* and *abd-A*, which phenocopied *PRC2* mutants, such as *E(z)*^*731*^ (Additional file 5: Fig. S5) and *esc*^*21*^ [41]. In stark contrast to this, loss of PRC1 components, such as Ph and Psc-Su(z)2, led to de-silencing of Abd-B and Scr, but not Ubx, in ddaC neurons (Fig. 4). Second, the extent of *Scm* phenotypes largely resembles *PRC2* mutants in terms of their dendrite arbourization and severing defects (Figs. 1 and 3). The dendrite arbourization and severing defects in *Scm* mutant or RNAi ddaC neurons were relatively weak, as compared to those in *ph* and *Psc-Su(z)2* (*PRC1*) mutants (Figs. 1 and 2). Finally, Scm is dispensable for repression of Abd-B in MB γ neurons (Additional file 9: Fig. S9), whereas the core PRC1 component Ph is important for Abd-B silencing (Fig. 7). Thus, these data suggest that Scm likely acts as a part of PRC2 in ddaC and MB γ neurons during pruning. In support of this notion, a recent study has reported a direct interaction between Scm and several core PRC2 components [60]. Moreover, Scm is also required for proper localization of the core PRC2 component E(z) on polytene chromosomes [60].

### PRC1 regulates neuronal pruning independent of PRC2

In the canonical model, PRC1 and PRC2 function cooperatively to silence their common target genes. The histone methyltransferase E(z) in the PRC2 complex tri-methylate histone H3 lysine 27 (H3K27) on the nucleosomes of the target genes, which is subsequently recognized by Pc in the PRC1 complex for chromatin compaction and transcriptional repression [35, 36]. Interestingly, our data support the non-canonical model in which PRC1 and PRC2 play independent roles in regulating dendrite pruning. PRC1 and PRC2 repress different Hox genes in ddaC neurons. PRC1 silences Abd-B and Scr expression (Fig. 4), whereas PRC2 represses Ubx level (Additional file 5: Fig. S5) [41]. These data suggest separable roles of PRC1 and PRC2. In line with this possibility, the dendrite pruning phenotype was enhanced in double mutant neurons of *E(z)*^*731*^ and *Pc*^*15*^, as compared to their individual mutants (Fig. 3). Moreover, PRC1 appears to play more important roles than PRC2 in ddaC sensory neurons. First, loss of PRC1 severely impaired larval dendrite arbourization and caused the dendrite severing defects with higher penetrance (Fig. 2), whereas *PRC2* mutants largely showed the dendrite fragmentation defects (Fig. 3), a relatively mild pruning phenotype. Second, while the PRC1 targets, Abd-B and Scr, were completely silenced in wild-type ddaC neurons (Additional file 4: Fig. S4A), they were drastically elevated in *PRC1* mutant neurons (Fig. 4). In contrast, the PRC2 targets, Ubx and Abd-A, were expressed at low levels in wild-type ddaC neurons (Additional file 4: Fig. S4A), indicative of mild repression. Loss of PRC2 components led to weak but significant elevations in Ubx and Abd-A protein levels (Additional file 5: Fig. S5). Third, overexpression of the major PRC1 target Abd-B caused the strongest dendrite pruning defects among the Hox proteins examined (Fig. 5). Interestingly, the previous studies in fly wing discs and brains have also reported Abd-B as an important target of PRC1 components, Ph, Pc and/or Psc/Su(z)2 [42, 51]. In addition, we also found that the key PRC1 component Ph is required for silencing of Abd-B in MB γ neurons (Fig. 7), whereas Scm is dispensable for Hox gene repression in those neurons (Additional file 9: Fig. S9). These data further strengthen the notion that PRC1 acts independently of the PRC2 complex. Consistent with the PRC2-independent function of PRC1, recent studies have reported that in fly imaginal discs, PRC1 can target a large set of genes that do not contain the PRC2-dependent repressive marker H3K27me3 [74]; moreover, PRC1 alone is sufficient for both activation and suppression of various target genes [74–76].

### Repression of Hox genes requires different PcG genes in the remodelling neurons

It has long been thought that the repressive state of classic PcG targets require a coordination between both PRC1 and PRC2 complexes (Kassis et al., 2017). In this study, our data indicate that in post-mitotic neurons, the repression of each Hox gene requires different PcG proteins. For instance, the silencing of Abd-B in larval ddaC neurons requires PRC1 components, such as Ph, Psc-Su(z)2, and Pc (Fig. 4), but not the PRC2 components E(z) (Additional file 5: Fig. S5) or Scm (Additional file 6: Fig. S6), whereas the repression of Ubx requires E(z) and Scm (Additional file 5–6: Fig. S5-6). However, the repression of Abd-A requires both PRC1 and PRC2 components (Fig. 4, Additional file 5–6: Fig. S5-6). Likewise, it has also been reported that in fly brains, Abd-B repression also requires Ph and Pc but not E(z), whereas the repression of another Hox protein Antp requires Pc and E(z) but not Ph (Wang et al., 2006). Moreover, silencing of Hox genes by PcG proteins appears to be context-dependent, which varies in different types of neurons. The silencing of Scr requires Ph in the peripheral ddaC neurons (Fig. 4) but not in the central MB γ neurons (Fig. 7). Despite the distinct regulatory mechanisms of Hox gene repression, derepressed Hox genes negatively regulate neuronal pruning, as their overexpression caused defective dendrite/axon pruning phenotypes in both ddaC neurons and/or MB γ neurons (Figs. 5 and 7).

### Ph and Abd-B are involved in activation of ecdysone signalling

In the previous studies, we identified two downstream targets of ecdysone signalling, such as the transcription factor Sox14 and the F-actin disassembly factor Mical, which play important roles in dendrite pruning of ddaC neurons [27]. In response to the late 3rd ecdysone pulse, the neuronal isoform EcR-B1 is upregulated in ddaC and interacts with the histone acetyltransferase (CBP). The chromatin remodeller Brm facilitates the interaction between EcR-B1 and CBP, presumably via modifying chromatin accessibility of the *sox14* gene locus. EcR-B1 acts together with Brm and CBP to promote local acetylation of H3K27 (H3K27Ac) around the *sox14* gene to activate its expression [29]. Sox14 is a rate-limiting factor that determines the initiation timing of dendrite pruning, as its overexpression can accelerate the progression of dendrite pruning as well as premature expression of Mical [27]. The mechanism underlying Sox14 expression has been well investigated. However, whether and how Mical expression is tightly regulated remain less understood. A previous study has reported that the eIF3-eIF4A-dependent translational initiation pathway is required for the translation of Mical protein [30]. Here, we report that Ph knockdown and Abd-B overexpression specifically downregulated the protein levels of Mical, but not EcR-B1 and Sox14. Ph and Abd-B likely regulate Mical expression via transcriptional regulation. Since Abd-B was derepressed upon Ph depletion, increased Abd-B may modify the chromatin landscape to reduce chromatin accessibility around the *mical* locus, thereby inhibiting Mical expression. Alternatively, Hox proteins are major drivers of gene transcriptional repression [77]. Abd-B may associate with a transcriptional repressive complex to repress *mical* transcription and thereby inhibit dendrite pruning. Unexpectedly, knockdown of Abd-B in *ph* RNAi ddaC neurons neither restored Mical levels (Additional file 7: Fig. S7C) nor rescued the dendrite pruning defects (Additional file 7: Fig. S7A). A possible explanation is that Ph may regulate Mical expression directly or indirectly through multiple targets. In line with this, growing evidence has showed that PRC1 complex is sufficient to activate and suppress a large number of genes [74–76].

## Conclusions

Our work emphasizes new and essential functions of PcG and Hox genes in regulating ecdysone signalling and neuronal pruning in *Drosophila*. Moreover, this study also suggests a non-canonical and PRC2-independent role of PRC1 in Hox gene silencing during neuronal pruning. Given that PcG, Hox genes, Mical and EcR/Sox14 are highly conserved in mammals including humans, our study would pave the way for future studies of their involvement in the pruning of mammalian nervous systems.

## Methods

### Fly strains

All *Drosophila* stocks and crosses were maintained in standard cornmeal media at 25 °C. The third instar larvae or early pupae at 0, 6, 16, 20 or 24 h APF (both male and female) were used in this study. The following stocks were requested from other labs: *UAS-Mical*^*N−ter*^ (non-functional N-terminal Mical fragment as a *UAS-control* transgene), *UAS-Mical*^*FL*^ [78], *ppk-Gal4* [79], *SOP-flp* [80], *71G10-Gal4* [23], *mhc-Gal80* [81], *Scm*^*D1*^, *Scm*^*M56*^ [43], *ph*^*505*^ [37, 48], *E(z)*^*73*^ [82].

The following stocks were obtained from Bloomington *Drosophila* Stock Center (BDSC): *UAS-mCD8-GFP, UAS-Dicer2, tubP-Gal80, FRT19A, FRT42D, FRT2A, FRT82B, GSG2295-Gal4* (BL#40,266), *ppk-CD4-tdGFP* (BL#35,843), *201Y-Gal4* (BL#*4440*), ctrl RNAi (mCherry, BL#35,785), *Df(3R)by10* (BL#1931), *Df(3R)BSC468* (BL#24,972), *Pc*^*15*^ (BL#24,468), *E(z)*^*731*^ (BL#24,470), *Su(z)12*^*2*^ (BL#24,159), *Su(z)12*^*4*^ (BL#24,469), *Scm* RNAi #1 (BL#55,278), *Scm* RNAi #2 (BL#35,389)*, Scm* RNAi #3 (BL#31,614), *Psc-Su(z)2*^*1.b8*^ (BL#24,467), *Psc* RNAi (BL#35,297), *Psc* RNAi (BL#31,611), *Psc* RNAi (BL#38,261), *Sce* RNAi (BL#35,446), *Sce* RNAi (BL#31,612), *ph-d* RNAi (BL#63,018), *ph-d* RNAi (BL#31,190), *ph-p* RNAi (BL#35,207), *ph-p* RNAi (BL#33,669), *ph-p* RNAi (BL#31,608), *Pc* *RNAi* (*BL#31,110*), *Psc-Su(z)2*^*1.b8*^ (BL#24,467), *esc*^*21*^ (BL#3623), *Df(2L)Exel6030* (BL#7513), *UAS-Abd-B* (BL#913)*, UAS-abd-A* (BL#912)*, UAS-Ubx* (BL#911), *UAS-Scr* (BL#7302), *Abd-B* RNAi (BL#26,746), *Scr* RNAi (BL#50,662).

The following stocks were obtained from Vienna *Drosophila* Resource Centre (VDRC): *ph* RNAi (v50028), *Su(z)2* RNAi (v50368), *Sce* RNAi (v106328), *Su(z)2* RNAi (v100096), control RNAi (v25271, *γ-tub37C*).

Genotypes of the fly strains shown in each figure are listed in Supplementary Methods.

### Immunohistochemistry and antibodies

The following antibodies were used for immunohistochemistry in this study: mouse anti-Scr (1:50; 6H-4.1, DSHB), mouse anti-Ubx (1:40; FP3-38, DSHB), mouse anti-Abd-A (1:100; 6A8.12, DSHB), mouse anti-Abd-B (1:40; 1A2E9, DSHB), mouse anti-EcR-B1 (1:50; AD4.4, DSHB), mouse anti-FasII (1:50; 1D4, DSHB), mouse anti-Cut (1:50; 2B10, DSHB), mouse anti-Knot/Collier (1:100, a gift from A. Vincent), Guinea pig anti-Sox14 (1:200), Guinea pig anti-Mical (1:500), Rabbit anti-GFP (1:1000, A-11122, Invitrogen), Rabbit anti-Scm (1:20; a gift from J. Muller). Fluorescein isothiocyanate (FITC)-, Cy3- and Cy5-conjugated secondary antibodies (111–545-003, 115–165-003, 111–165-003, 106–165-003 and 123–605-021, Jackson ImmunoResearch) were used at 1:500 dilution.

For immunohistochemistry, pupae or larvae were dissected in cold PBS and fixed in 4% formaldehyde for 20 min, followed by washing with 0.5% Triton-X-containing PBS (PBST) for 3 times. Control and sample fillets/brains for each set of experiment were washed and stained in the same tube. Primary antibodies were added into blocking buffer (5% BSA in PBST) after 30 min blocking and were incubated at 4 °C overnight. Secondary antibodies were incubated on the second day at room temperature for 2–6 h. Samples were mounted using VectaShield mounting medium and imaged using either Leica SPE II or Olympus FV3000 confocal microscope. Images were taken from projected z-stacks (1.5-µm intervals) to cover the whole da neuron. Images of the same experiment set were taken with the same settings and processed in parallel.

Measurement of fluorescence intensity was done using ImageJ. Contours of cell nuclei (Ubx/ Abd-A/ Abd-B/ Scr/ EcR-B1/ Sox14/ Cut/ Knot immunostaining) or whole soma (Mical immunostaining) were drawn on the GFP channel. To quantify the fluorescence intensity of Scr, Cut and Knot background (rolling ball radius = 50) was subtracted on the whole image of that channel before measuring the mean grey value in the marked region of ddaC nuclei was measured. To quantify the fluorescence intensity of Ubx, Abd-A, EcR-B1, Sox14 and Mical, background (rolling ball radius = 50) was subtracted on the whole image of that channel before measuring the mean grey value in the marked region of ddaC and ddaE, their ratio was subsequently calculated. The values were then normalized to their corresponding mean control values and subjected to statistical analysis.

### Mosaic analysis with a repressible cell marker (MARCM) and RNAi analysis of da neurons

MARCM and RNAi analyses of da neurons’ dendrites were carried out as previously described (Kirilly et al., 2009). To image da neurons of WP, pupae was washed briefly in PBS before being mounted onto slides with 90% glycerol. To image da neurons at 16 h APF, pupae were collected onto moist filter paper and left overnight at 25 °C. After 16 h, pupae case was removed carefully and mounted onto slides with 90% glycerol. Live confocal images of da neurons expressing mCD8-GFP was taken with Leica SPE II confocal microscope. Dorsal is up in all images.

To quantify the pruning defects of ddaC neurons, percentages of severing and fragmentation defects were calculated in a 275 µm × 275 µm region of dorsal dendritic region (abdominal segments 2–4). Severing defect is defined as neurons with dendrites still attached to the soma. Fragmentation defect is defined as the presence of dendrites near the ddaC dorsal region but have been severed at the proximal regions to the soma. Total length of unpruned dendrites was measured using the ImageJ plugin, simple neurite tracer and scatter plots were generated using Graphpad Prism software. Sholl analysis of dendrite morphology was conducted using ImageJ. Plots of average length, number of intersections and SEM were generated using Graphpad Prism software.

### RU486 treatment for GeneSwitch experiments

Embryos were collected at 12-h intervals and reared on normal food until 2nd instar larvae (about 72 h after egg laying AEL). The larvae were transferred to food containing 240 µg/ml RU486/mifepristone. After 48 h, white pupae were collected for further analysis.

### Statistical analysis

For pairwise comparisons, two-tailed Student’s *t* test was applied to determine statistical significance. One-way ANOVA with Bonferroni test was applied when multiple groups were present. Statistical significance is as defined, *** *p* < 0.001, **p < 0.01, *p < 0.05, ns, not significant. Standard error of the mean (SEM) is indicated in the error bars of all graphs. The number of neurons (*n*) in each group is shown on the bars. All quantitative data are included in Additional file 11: Table S1.

## Supplementary Information

**Additional file 1: Figure S1.** Scm is required for dendrite pruning in ddaC neurons.

**Additional file 2: Figure S2.** *Scm* is required for dendrite pruning of class I ddaD/ddaE neurons but not for apoptosis of class III ddaF neurons.

**Additional file 3: Figure S3.** PRC1 is required for dendrite pruning in ddaC neurons.

**Additional file 4: Figure S4.** Ph silences Scr expression in ddaC neurons.

**Additional file 5: Figure S5.** The PRC2 component E(z) is required for suppression of Ubx and Abd-A expression in ddaC neurons.

**Additional file 6: Figure S6.** Scm is required for suppression of Ubx and Abd-A expression in ddaC neurons.

**Additional file 7: Figure S7.** Knockdown of Abd-B or Scr did not rescue the dendrite pruning defects in *ph *RNAi ddaC neurons.

**Additional file 8: Figure S8.** PRC1, but not PRC2, is important for Mical expression in ddaC neurons before pruning.

**Additional file 9: Figure S9.** Scm is required for axonal pruning in MB γ neurons.

**Additional file 10: Figure S10.** A schematic representation summarizes the potential role of Ph and Abd-B proteins in regulating Mical expression and thereby ecdysone signalling during dendrite pruning.

**Additional file 11: Table S1.** Source data for all the figures.

**Additional file 12: **A list of fly strains used in all the figures.

**Additional file 13: **Legends for Additional files 1–12.

## Data Availability

All data generated or analysed during this study are included in this published article and its supplementary information files. The raw microscopy datasets are available from the corresponding author on reasonable request. All quantitative data are included in Additional file 11: Table S1.

## Abbreviations

dda: Dorsal dendritic arbourization
MB: Mushroom body
PcG: Polycomb group
PRC1: Polycomb repressive complex 1
PRC2: Polycomb repressive complex 2
wL3: Wandering third instar larval
WP: White prepupal
APF: After puparium formation
EcR: Ecdysone receptor
Usp: Ultraspiracle
CBP: CREB-binding protein
Scm: Sex comb on midleg
Ph: Polyhomeotic
Pc: Polycomb
Psc: Posterior Sex Combs
Su(z)2: Suppressor of zeste 2
Su(z)12: Suppressor of zeste 12
MARCM: Mosaic analysis with a repressive cell marker
Ubx: Ultrabithorax
Abd-A: Abdominal A
Abd-B: Abdominal B
