# Supplementary figures and images for: Polycomb group genes are required for neuronal pruning in Drosophila
Source: BMC Biol. 2023 Feb 15;21:33. doi: 10.1186/s12915-023-01534-0 (PMC9933400; doi:10.1186/s12915-023-01534-0)

# Figure S1

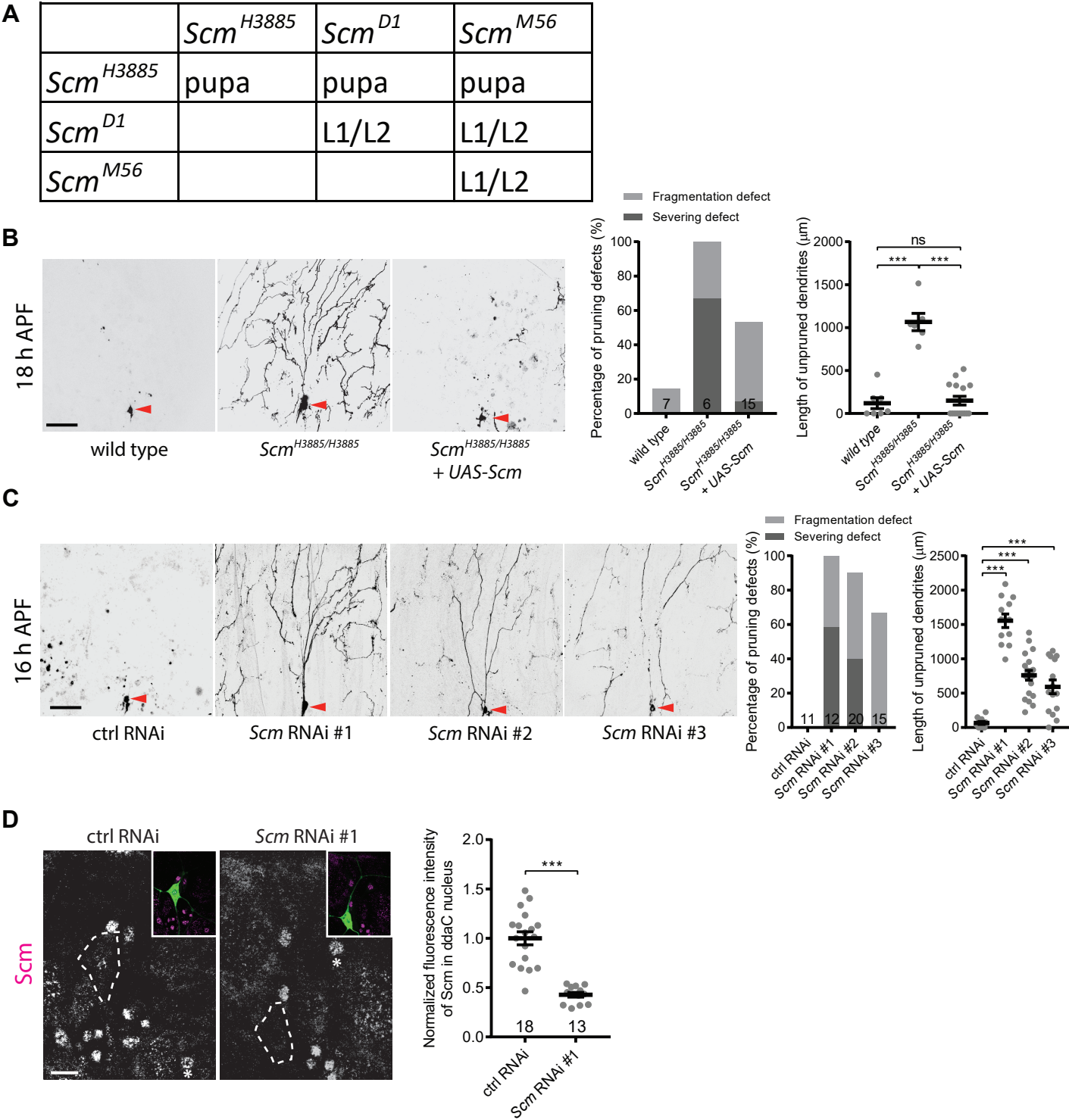

Supplement: Supplementary file 1 — Additional file 1: Figure S1. Scm is required for dendrite pruning in ddaC neurons. [file 12915_2023_1534_MOESM1_ESM.pdf]

# Figure S2

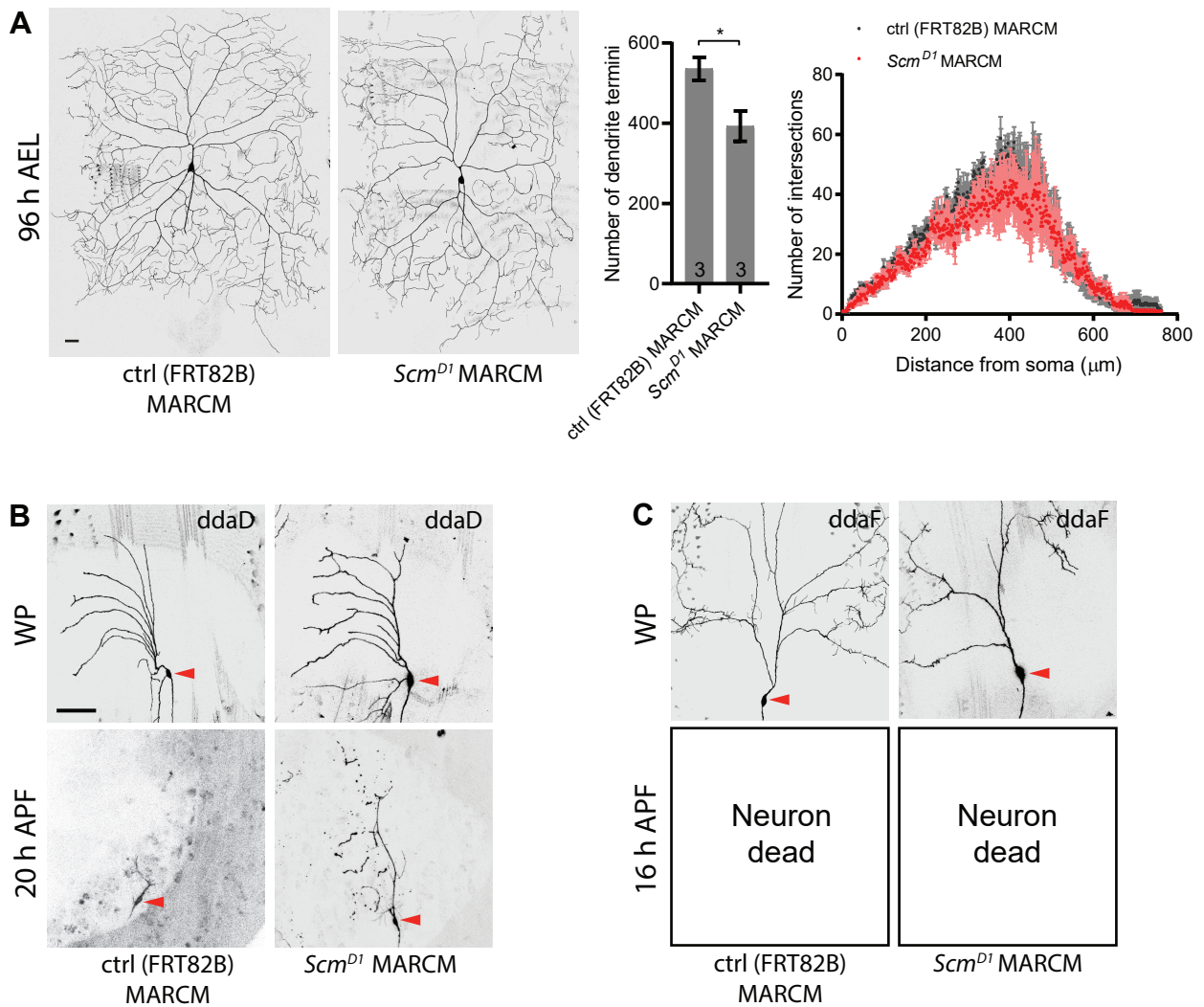

Supplement: Supplementary file 2 — Additional file 2: Figure S2. Scm is required for dendrite pruning of class I ddaD/ddaE neurons but not for apoptosis of class III ddaF neurons. [file 12915_2023_1534_MOESM2_ESM.pdf]

# Figure S3

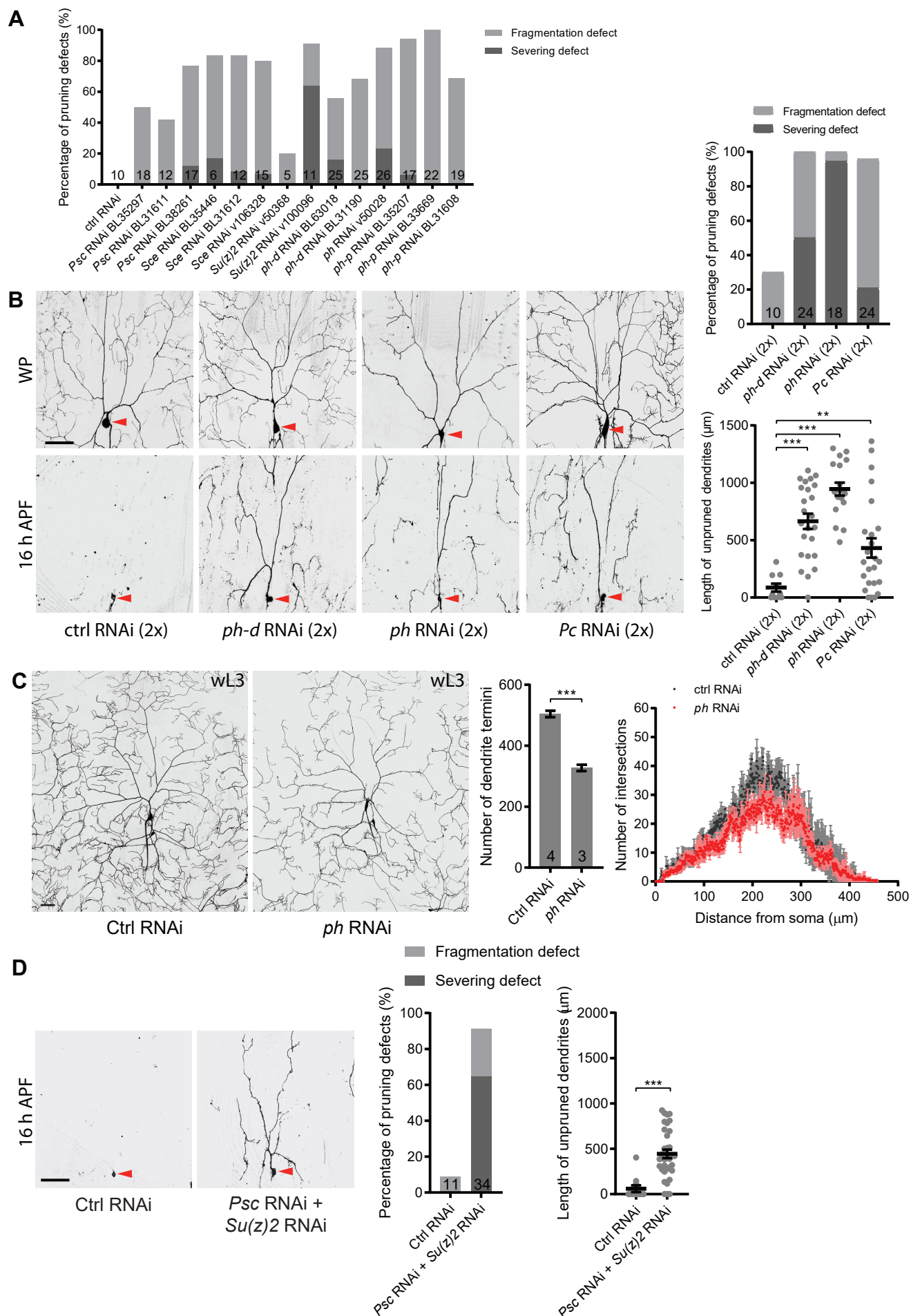

Supplement: Supplementary file 3 — Additional file 3: Figure S3. PRC1 is required for dendrite pruning in ddaC neurons. [file 12915_2023_1534_MOESM3_ESM.pdf]

# Figure S4

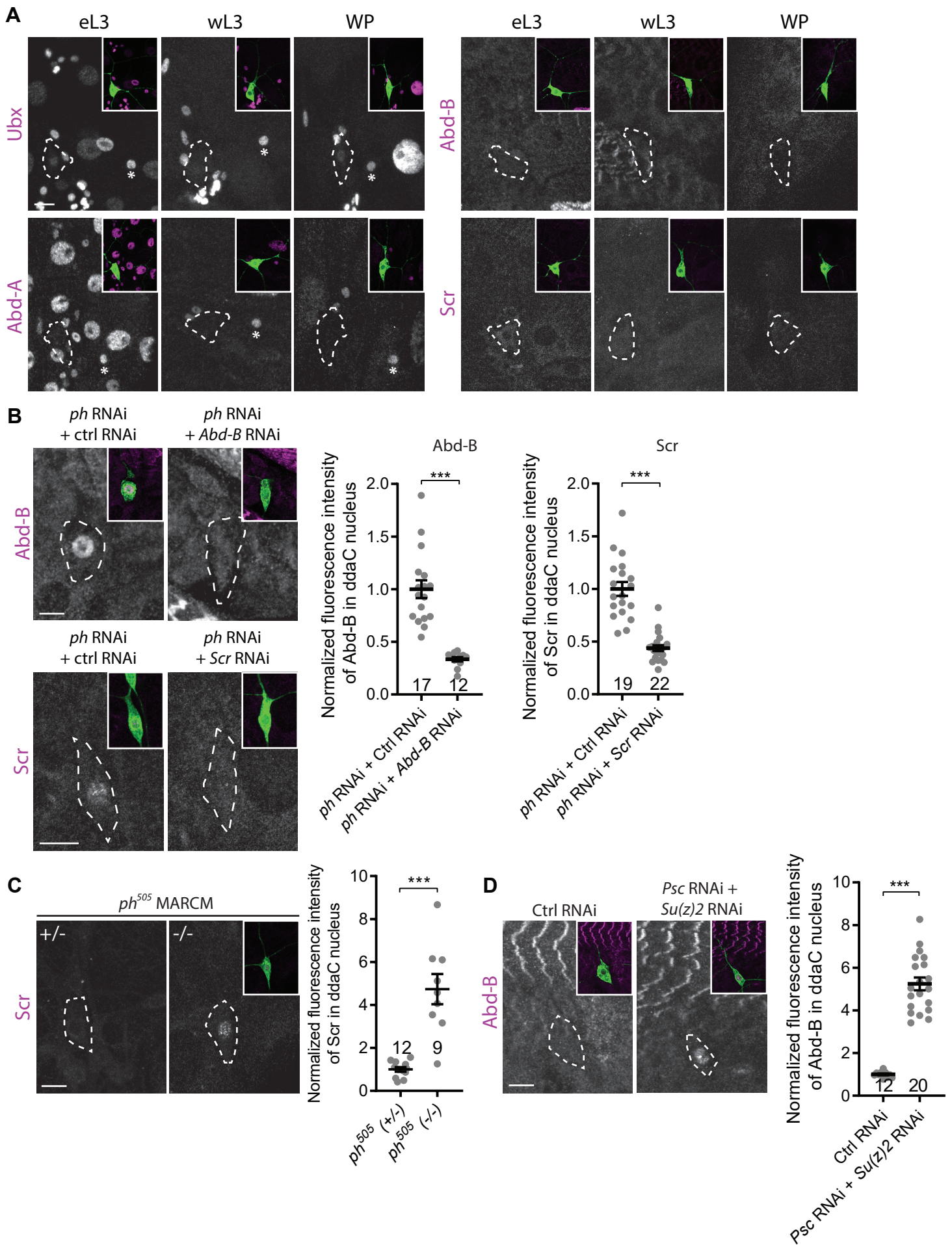

Supplement: Supplementary file 4 — Additional file 4: Figure S4. Ph silences Scr expression in ddaC neurons. [file 12915_2023_1534_MOESM4_ESM.pdf]

## Figure S5

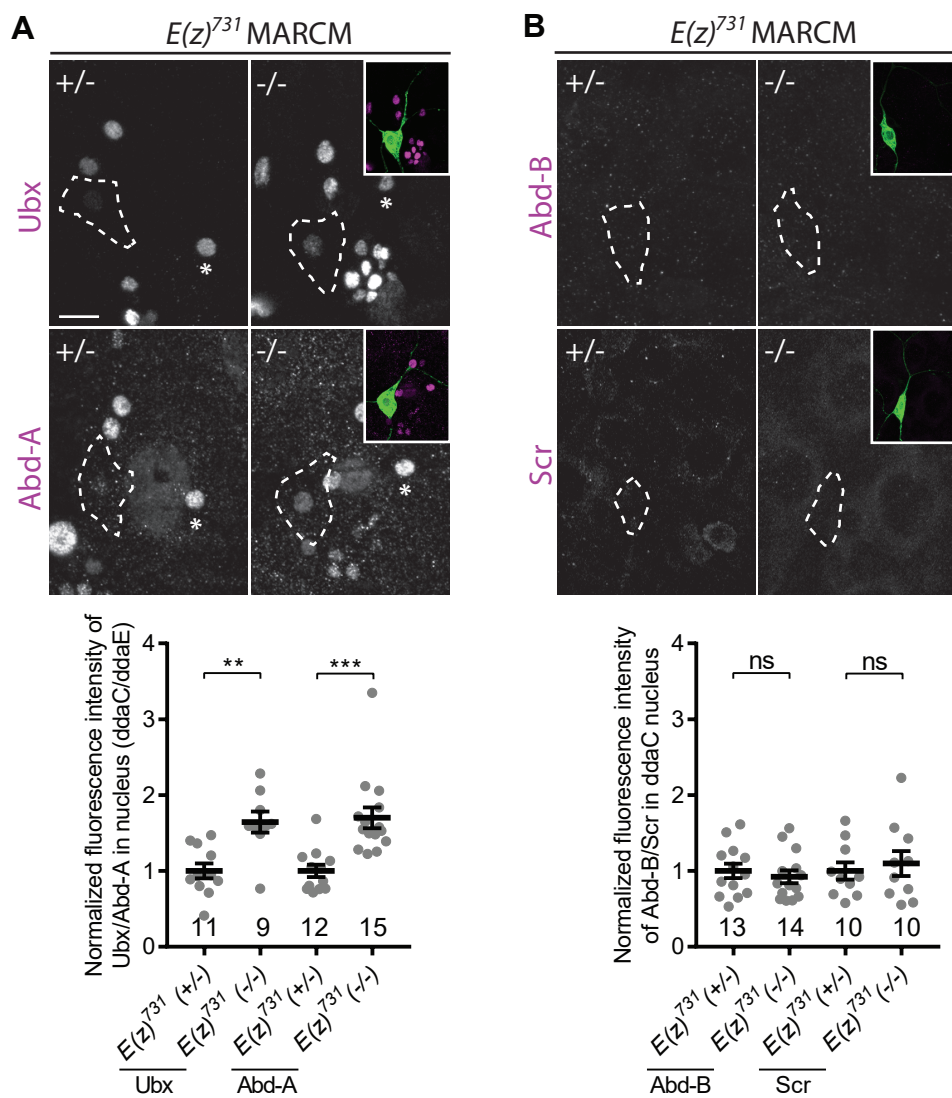

Supplement: Supplementary file 5 — Additional file 5: Figure S5. The PRC2 component E(z) is required for suppression of Ubx and Abd-A expression in ddaC neurons. [file 12915_2023_1534_MOESM5_ESM.pdf]

# Figure S6

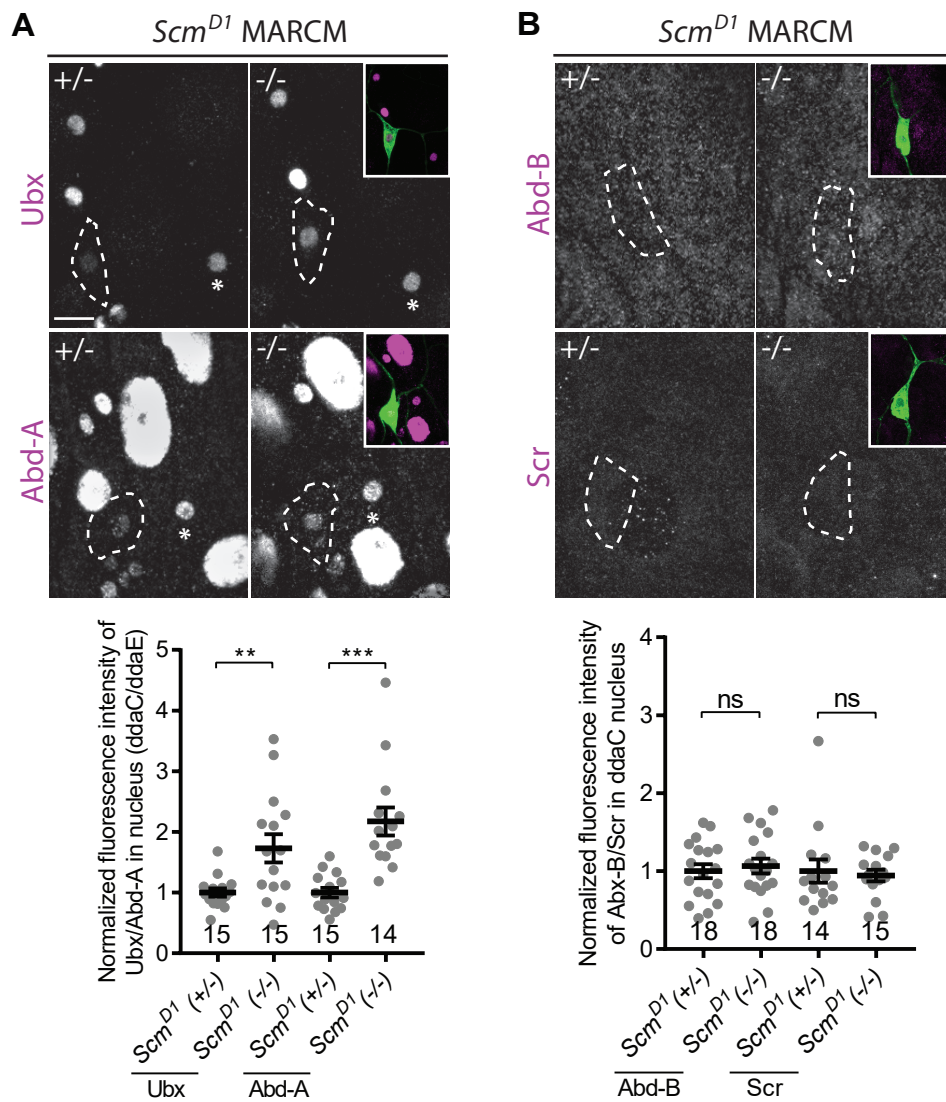

Supplement: Supplementary file 6 — Additional file 6: Figure S6. Scm is required for suppression of Ubx and Abd-A expression in ddaC neurons. [file 12915_2023_1534_MOESM6_ESM.pdf]

# Figure S7

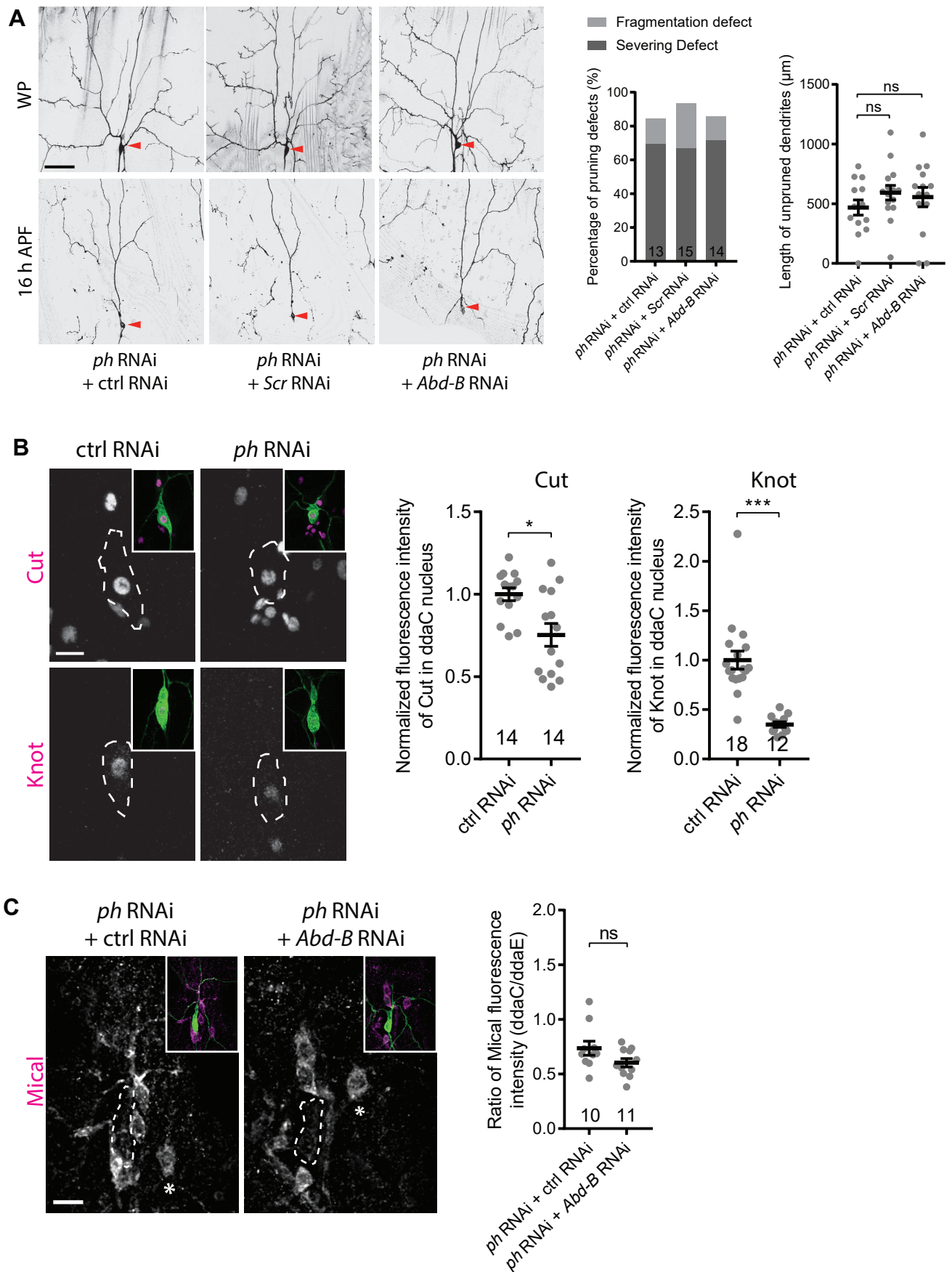

Supplement: Supplementary file 7 — Additional file 7: Figure S7. Knockdown of Abd-B or Scr did not rescue the dendrite pruning defects in ph RNAi ddaC neurons. [file 12915_2023_1534_MOESM7_ESM.pdf]

# Figure S8

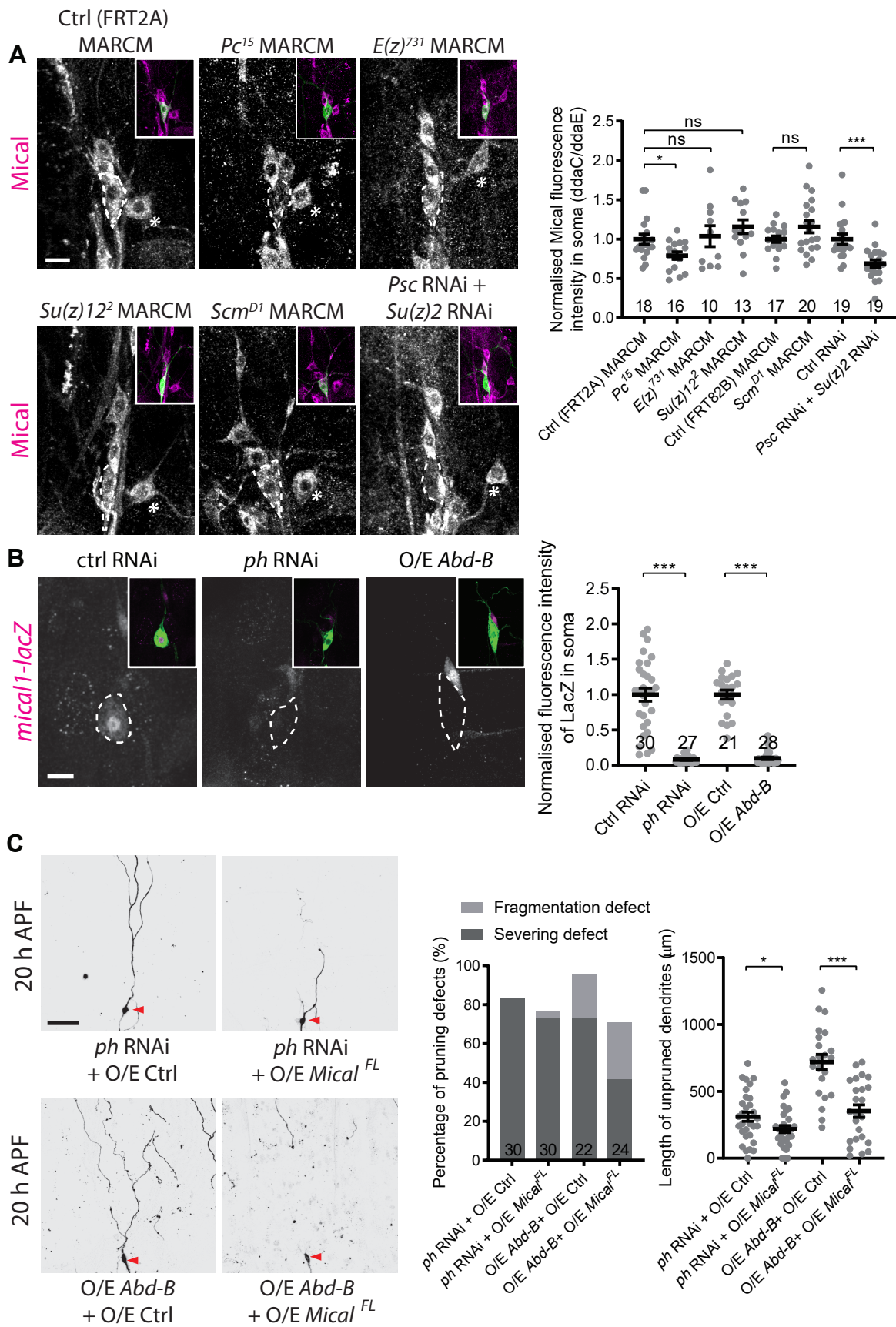

Supplement: Supplementary file 8 — Additional file 8: Figure S8. PRC1, but not PRC2, is important for Mical expression in ddaC neurons before pruning. [file 12915_2023_1534_MOESM8_ESM.pdf]

# Figure S9

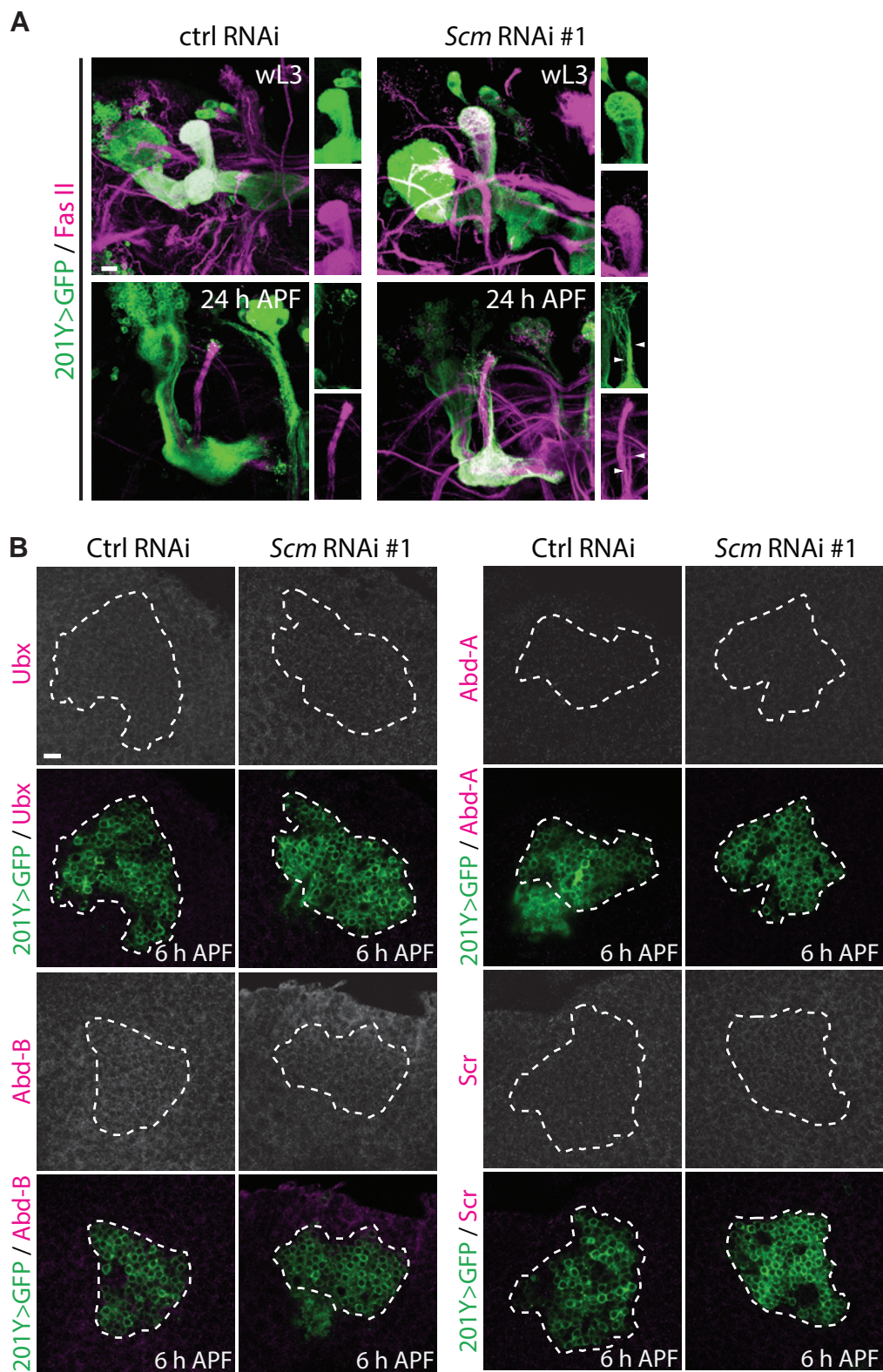

Supplement: Supplementary file 9 — Additional file 9: Figure S9. Scm is required for axonal pruning in MB γ neurons. [file 12915_2023_1534_MOESM9_ESM.pdf]

Figure S10

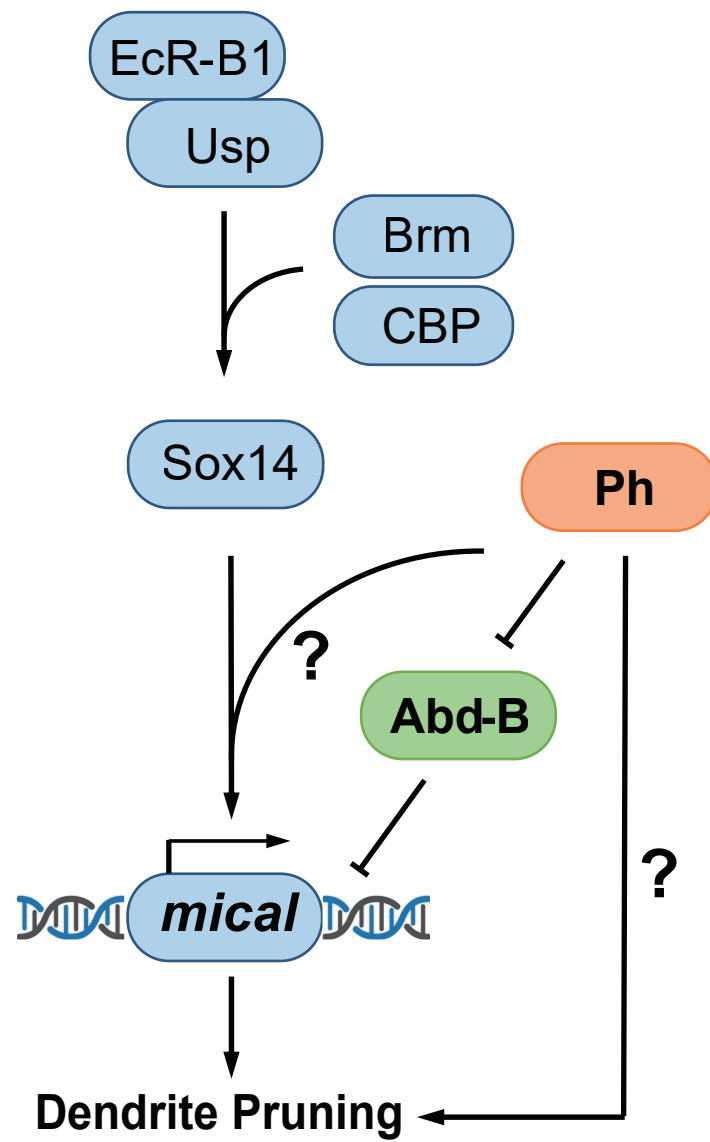

Supplement: Supplementary file 10 — Additional file 10: Figure S10. A schematic representation summarizes the potential role of Ph and Abd-B proteins in regulating Mical expression and thereby ecdysone signalling during dendrite pruning. [file 12915_2023_1534_MOESM10_ESM.pdf]
