# Supplementary material for: Polycomb group genes are required for neuronal pruning in Drosophila
Source: BMC Biol. 2023 Feb 15;21:33. doi: 10.1186/s12915-023-01534-0 (PMC9933400; doi:10.1186/s12915-023-01534-0)
Supplement: Supplementary file 12 — Additional file 12: A list of fly strains used in all the figures. [file 12915_2023_1534_MOESM12_ESM.docx]

**A list of fly strains**

**Figure 1:**

(C) *w*; ppk-Gal4, UAS-mCD8-GFP/ ppk-Gal4, UAS-mCD8-GFP; +/+.*

(D) *w*; ppk-Gal4, UAS-mCD8-GFP/ ppk-Gal4, UAS-mCD8-GFP; Scm^H3885^ / Scm^H3885^.*

(E) *w*; ppk-Gal4, UAS-mCD8-GFP/+ ; Scm^H3885^ / FRT82B Scm^D1^.*

(F) *w*; ppk-Gal4, UAS-mCD8-GFP/+ ; Scm^H3885^ / FRT82B, Scm^M56^.*

(G) *w*; ppk-Gal4, UAS-mCD-GFP, SOP-flp / + ; FRT82B, tubP-Gal80 / FRT82B, Scm^D1^.*

(H) *w*; ppk-Gal4, UAS-mCD-GFP, SOP-flp / + ; FRT82B, tubP-Gal80 / FRT82B, Scm^M56^.*

**Figure 2:**

(A) *w*; ppk-Gal4, UAS-mCD8-GFP / + ; ctrl* *RNAi / UAS-Dcr2.*

(B) *w*; ppk-Gal4, UAS-mCD8-GFP / + ; ph RNAi / UAS-Dcr2.*

(C) *w*, FRT19A, tubP-Gal80 / w*, FRT19A, ph^505^; ppk-Gal4, UAS-mCD8-GFP, SOP-flp / +.*

(D) *w*, FRT19A, tubP-Gal80 / w*, FRT19A, ph^505^; ppk-Gal4, UAS-mCD8-GFP, SOP-flp / +; UAS-ph-p / +.*

(E, F) *w*; GSG2295-Gal4, ppk-tdGFP/+ ; UAS-Dcr2/ ph RNAi.*

(G) *w*; ppk-Gal4, UAS-mCD8-GFP, SOP-flp / + ; FRT2A, tubP-Gal80 / FRT2A, Pc^15^.*

(H) *w*; FRT42D, tubP-Gal80 / FRT42D, Psc-Su(z)2^1.b8^; ppk-Gal4, UAS-mCD8-GFP, SOP-flp / +.*

**Figure 3:**

(A) *w**; *ppk-Gal4, UAS-mCD8-GFP, SOP-flp* / *+ ; FRT2A* / *FRT2A, tubP-Gal80.*

(B) *w*; ppk-Gal4, UAS-mCD8­-GFP, SOP-flp* / *+ ; FRT2A, E(z)^73^* / *FRT2A, tubP-Gal80.*

(C) *w*; ppk-Gal4, UAS-mCD8­-GFP, SOP-flp* / *+ ; FRT2A, E(z)^731^* / *FRT2A, tubP-Gal80.*

(D) *w*; ppk-Gal4, UAS-mCD8­-GFP, SOP-flp* / *+ ; FRT2A, Su(z)12^2^* / *FRT2A, tubP-Gal80.*

(E) *w*; ppk-Gal4, UAS-mCD8­-GFP, SOP-flp* / *+ ; FRT2A, Su(z)12^4^* / *FRT2A, tubP-Gal80.*

(F) *w*; esc^21^* / *Df (2L) Exel 6030; ppk-Gal4, UAS-mCD8-GFP* / *+.*

(I) *w*; ppk-Gal4, UAS-mCD8-GFP, SOP-flp / + ; FRT2A, Pc^15^/ FRT2A, tubP-Gal80.*

(J) *w*; ppk-Gal4, UAS-mCD8-GFP, SOP-flp / + ; FRT2A, Pc^15^, E(z)^731^ / FRT2A, tubP-Gal80.*

**Figure 4:**

(A) **ctrl RNAi:** *w*; ppk-Gal4, UAS-mCD8-GFP, UAS-Dcr2 / ppk-Gal4, UAS-mCD8-GFP, UAS-Dcr2; ctrl RNAi / ctrl RNAi.* ***ph* RNAi:** *w*; ppk-Gal4, UAS-mCD8-GFP, UAS-Dcr2 / ppk-Gal4, UAS-mCD8-GFP, UAS-Dcr2; ph RNAi / ph RNAi.*

(C) ***ph^505^* MARCM:** *w*, FRT19A, tubP-Gal80 / w*, FRT19A, ph^505^; ppk-Gal4, UAS-mCD8-GFP, SOP-flp / +.* ***Pc^15^* MARCM:** *w*; ppk-Gal4, UAS-mCD8-GFP, SOP-flp / + ; FRT2A, tubP-Gal80 / FRT2A, Pc^15^.* ***Psc-Su(z)2^1.b8^* MARCM:** *w*; FRT42D, tubP-Gal80 / FRT42D, Psc-Su(z)2^1.b8^; ppk-Gal4, UAS-mCD8-GFP, SOP-flp / +.*

**Figure 5:**

(A) *w*; ppk-Gal4 /* *+ ; ppk-Gal4, UAS-mCD-8GFP /* *UAS-Mical^N-ter^.*

(B) *w*; ppk-Gal4 / + ; ppk-Gal4, UAS-mCD8-GFP / UAS-Ubx.*

(C) *w*; ppk-Gal4 / + ; ppk-Gal4, UAS-mCD8-GFP / UAS-abd-A.*

(D) *w*; ppk-Gal4 / + ; ppk-Gal4, UAS-mCD8-GFP / UAS-Abd-B.*

(E) *w*; ppk-Gal4 / UAS-Scr; ppk-Gal4, UAS-mCD8-GFP / +.*

(F) *w*; GSG2295-Gal4, ppk-tdGFP / + ; UAS-Mical^N-ter^.*

(G) *w*; GSG2295-Gal4, ppk-tdGFP / + ; UAS-Ubx.*

(H) *w*; GSG2295-Gal4, ppk-tdGFP / + ; UAS-Abd-B.*

**Figure 6:**

(A-L) **ctrl RNAi:** *w*; ppk-Gal4, UAS-mCD8-GFP, UAS-Dcr2 / ppk-Gal4, UAS-mCD8-GFP, UAS-Dcr2; ctrl RNAi / ctrl RNAi.* ***ph* RNAi:** *w*; ppk-Gal4, UAS-mCD8-GFP, UAS-Dcr2 / ppk-Gal4, UAS-mCD8-GFP, UAS-Dcr2; ph RNAi / ph RNAi.* **O/E *abd-A*:** *w*; ppk-Gal4 / + ; ppk-Gal4, UAS-mCD8-GFP / UAS-abd-A.* **O/E *Abd-B*:** *w*; ppk-Gal4 / + ; ppk-Gal4, UAS-mCD8-GFP / UAS-Abd-B.*

**Figure 7:**

(A, D, F, H, J) *w*; ppk-Gal4, UAS-mCD8-GFP, UAS-Dcr2/ 201Y-Gal4, UAS-mCD8-GFP; ctrl RNAi / mhc-Gal80.*

(B, E, G, I, K) *w*; ppk-Gal4, UAS-mCD8-GFP, UAS-Dcr2/ 201Y-Gal4, UAS-mCD8-GFP; ph RNAi / mhc-Gal80.*

(C) *w*;; 71G10-Gal4, UAS-mCD8-GFP/ UAS-Abd-B.*

**Figure S1:**

(B) **wild type:** *w*; ppk-Gal4, UAS-mCD8­-GFP* / *+.* ***Scm^H3885^***^/^***^H3885^*:** *w*; ppk-Gal4, UAS-mCD8-GFP / + ; Scm^H3885^ / Scm^H3885^.* ***Scm^H3885^***^/^***^H3885^ + UAS-Scm*:** *w*; ppk-Gal4, UAS-mCD8-GFP* / *UAS-Scm; Scm^H3885^* / *Scm^H3885^*.

(C) **ctrl RNAi:** *w*; ppk-Gal4, UAS-mCD8-GFP* / *+ ; UAS-Dcr2* / *ctrl RNAi.* ***Scm* RNAi #1:** *w*; ppk-Gal4, UAS-mCD8-GFP* / *Scm RNAi* (*BL55278*)*; UAS-Dcr2* / *+*. ***Scm* RNAi #2:** *w*; ppk-Gal4, UAS-mCD8-GFP* / *+ ; UAS-Dcr2* / *Scm RNAi* (*BL35389*). ***Scm* RNAi #3:** *w*; ppk-Gal4, UAS-mCD8-GFP* / *+ ; UAS-Dcr2* / *Scm RNAi* (*BL31614*).

(D) **ctrl RNAi:** *w*; ppk-Gal4 / +; ctrl RNAi* / *ppk-Gal4, UAS-mCD8­-GFP.* ***Scm* RNAi #1:** *w*; ppk-Gal4* / *Scm RNAi* (*BL55278*)*; ppk-Gal4, UAS-mCD8-GFP* / *+*.

**Figure S2:**

(A) **Ctrl (FRT82B) MARCM:** *w*; ppk-Gal4, UAS-mCD8­-GFP, SOP-flp* / *+ ; FRT82B* / *FRT82B, tubP-Gal80.* ***Scm^D1^* MARCM**: *w*; ppk-Gal4, UAS-mCD8­-GFP, SOP-flp* / *+ ; FRT82B, Scm^D1^* / *FRT82B, tubP-Gal80.*

(B, C) **Ctrl (FRT82B) MARCM:** *w*; Gal4^109(2)80^, UAS-mCD8­-GFP, SOP-flp* / *+ ; FRT82B* / *FRT82B, tubP-Gal80.* ***Scm^D1^* MARCM:** *w*; Gal4^109(2)80^, UAS-mCD8-­GFP, SOP-flp* / *+ ; FRT82B, Scm^D1^* / *FRT82B, tubP-Gal80.*

**Figure S3:**

(B) **ctrl RNAi (2x):** *w*; ppk-Gal4, UAS-mCD8-GFP, UAS-Dcr2* / *ppk-Gal4, UAS-mCD8-GFP, UAS-Dcr2; ctrl RNAi* / *ctrl RNAi.* ***ph-d* RNAi (2x):** *w*; ph-d RNAi* (*BL63018*) / *ph-d RNAi* (*BL63018*)*; ppk-Gal4, UAS-mCD8-GFP* / *ppk-Gal4, UAS-mCD8GFP.* ***ph* RNAi (2x):** *w*; ppk-Gal4, UAS-mCD8-GFP, UAS-Dcr2* / *ppk-Gal4, UAS-mCD8GFP, UAS-Dcr2; ph RNAi* (*v50028*) / *ph RNAi* (*v50028*)*.*  ***Pc* RNAi (2x):** *w*; ppk-Gal4, UAS-mCD8-GFP, UAS-Dcr2* / *ppk-Gal4, UAS-mCD8-GFP, UAS-Dcr2; Pc RNAi* (*BL31110*) / *Pc RNAi* (*BL31110*)*.*

(C) **ctrl RNAi:** *w*; ppk-Gal4, UAS-mCD8-GFP* / *+ ; ctrl RNAi* / *UAS-Dcr2.* ***ph* RNAi:** *w*; ppk-Gal4, UAS-mCD8-GFP*/ + *;* *ph RNAi* /  *UAS-Dcr2.*

(D) **ctrl RNAi:** *w*; ppk-Gal4, UAS-mCD8-GFP* / *+ ; ctrl RNAi* / *UAS-Dcr2.* ***Psc* RNAi + *Su(z)2* RNAi:** *w*; ppk-Gal4, UAS-mCD8-GFP* / *Su(z)2 RNAi (v100096) ; Psc RNAi (BL35297)* / *UAS-Dcr2.*

**Figure S4:**

(A) *w*;; ppk-Gal4, UAS-mCD8­-GFP* / *ppk-Gal4, UAS-mCD8­-GFP*.

(B) ***ph* RNAi *+ ctrl* RNAi:** *w*; ppk-Gal4, UAS-mCD8-GFP, UAS-Dcr2 / + ; ph RNAi / ctrl RNAi.* ***ph* RNAi *+ Abd-B* RNAi:** *w*; ppk-Gal4, UAS-mCD8-GFP, UAS-Dcr2 / + ; ph RNAi / Abd-B RNAi.* ***ph* RNAi *+ Scr* RNAi:** *w*; ppk-Gal4, UAS-mCD8-GFP, UAS-Dcr2 / + ; ph RNAi / Scr RNAi.*

(C) *w*, FRT19A, tubP-Gal80 / w*, FRT19A, ph^505^; ppk-Gal4, UAS-mCD8-GFP, SOP-flp / +.*

(D) **ctrl RNAi:** *w*; ppk-Gal4, UAS-mCD8-GFP* / *+ ; ctrl RNAi* / *UAS-Dcr2.* ***Psc* RNAi + *Su(z)2* RNAi:** *w*; ppk-Gal4, UAS-mCD8-GFP* / *Su(z)2 RNAi (v100096) ; Psc RNAi (BL35297)* / *UAS-Dcr2.*

**Figure S5:**

(A-B) *w*; ppk-Gal4, UAS-mCD8­-GFP, SOP-flp* / *+ ; FRT2A, E(z)^731^* / *FRT2A, tubP-Gal80.*

**Figure S6:**

(A-B) *w*; ppk-Gal4, UAS-mCD8­-GFP, SOP-flp* / *+ ; FRT82B, Scm^D1^* / *FRT82B, tubP-Gal80.*

**Figure S7:**

(A) ***ph* RNAi *+ ctrl* RNAi:** *w*; ppk-Gal4, UAS-mCD8-GFP, UAS-Dcr2 / + ; ph RNAi / ctrl RNAi.* ***ph* RNAi *+ Scr* RNAi:** *w*; ppk-Gal4, UAS-mCD8-GFP, UAS-Dcr2 / + ; ph RNAi / Scr RNAi.* ***ph* RNAi *+ Abd-B* RNAi:** *w*; ppk-Gal4, UAS-mCD8-GFP, UAS-Dcr2 / + ; ph RNAi / Abd-B RNAi.*

(B) **ctrl RNAi:** *w*; ppk-Gal4, UAS-mCD8-GFP* / *+ ; ctrl RNAi* / *UAS-Dcr2.* ***ph* RNAi:** *w*; ppk-Gal4, UAS-mCD8-GFP* / + *;* *ph RNAi* / *UAS-Dcr2*.

(C) ***ph* RNAi *+ ctrl* RNAi:** *w*; ppk-Gal4, UAS-mCD8-GFP, UAS-Dcr2 / + ; ph RNAi / ctrl RNAi.* ***ph* RNAi *+ Abd-B* RNAi:** *w*; ppk-Gal4, UAS-mCD8-GFP, UAS-Dcr2 / + ; ph RNAi / Abd-B RNAi.*

**Figure S8:**

(A) **Ctrl (FRT2A) MARCM:** *w**; *ppk-Gal4, UAS-mCD8-GFP, SOP-flp* / *+ ; FRT2A* / *FRT2A, tubP-Gal80.* ***Pc^15^* MARCM:** *w*; ppk-Gal4, UAS-mCD8-GFP, SOP-flp / + ; FRT2A, tubP-Gal80 / FRT2A, Pc^15^.* ***E(z)^731^* MARCM:** *w*; ppk-Gal4, UAS-mCD8­-GFP, SOP-flp* / *+ ; FRT2A, E(z)^731^* / *FRT2A, tubP-Gal80.* ***Su(z)12^2^* MARCM:** *w*; ppk-Gal4, UAS-mCD8­-GFP, SOP-flp* / *+ ; FRT2A, Su(z)12^2^* / *FRT2A, tubP-Gal80.* ***Scm^D1^* MARCM:** *w*; Gal4^109(2)80^, UAS-mCD8-­GFP, SOP-flp* / *+ ; FRT82B, Scm^D1^* / *FRT82B, tubP-Gal80.* ***Psc* RNAi + *Su(z)2* RNAi:** *w*; ppk-Gal4, UAS-mCD8-GFP* / *Su(z)2 RNAi (v100096) ; Psc RNAi (BL35297)* / *UAS-Dcr2.*

(B) **Ctrl RNAi:** *w*; ppk-Gal4, UAS-mCD8-GFP, UAS-Dcr2 / ppk-Gal4, UAS-mCD8-GFP, UAS-Dcr2; ctrl RNAi / mical1-lacZ.* ***ph* RNAi:** *w*; ppk-Gal4, UAS-mCD8-GFP, UAS-Dcr2 / ppk-Gal4, UAS-mCD8-GFP, UAS-Dcr2; ph RNAi / mical1-lacZ.* **O/E *Abd-B*:** *w*; ppk-Gal4, UAS-mCD8-GFP, UAS-Dcr2 / UAS-Abd-B; mical1-lacZ / +.*

(C) ***ph* RNAi + O/E Ctrl:** *w*; ppk-Gal4, UAS-mCD8-GFP, UAS-Dcr2 / ppk-Gal4, UAS-mCD8-GFP; ph-p RNAi / UAS-Control.* ***ph* RNAi + O/E *Mical^FL^*:** *w*; ppk-Gal4, UAS-mCD8-GFP, UAS-Dcr2 / ppk-Gal4, UAS-mCD8-GFP ; ph RNAi / UAS-Mical^FL^.* **O/E *Abd-B* + O/E Ctrl:** *w*; UAS-Abd-B / ppk-Gal4, UAS-mCD8-GFP; ppk-Gal4, UAS-mCD8-GFP / UAS-Control.* **O/E *Abd-B* + O/E *Mical^FL^*: *O/E Abd-B* + *O/E Mical^FL^:*** *w*; UAS-Abd-B / ppk-Gal4, UAS-mCD8-GFP; ppk-Gal4, UAS-mCD8-GFP / UAS- Mical^FL^.*

**Figure S9**

(A,B) **Ctrl RNAi:** *w*; ppk-Gal4, UAS-mCD8-GFP, UAS-Dcr2/ 201Y-Gal4, UAS-mCD8-GFP; ctrl RNAi / mhc-Gal80.* ***Scm* RNAi #1:** *w*; Scm RNAi* / *201Y-Gal4, UAS-mCD8-GFP; ppk-Gal4, UAS-mCD8-GFP, UAS-Dcr2 / mhc-Gal80*.
