# Supplementary material for: Polycomb group genes are required for neuronal pruning in Drosophila
Source: BMC Biol. 2023 Feb 15;21:33. doi: 10.1186/s12915-023-01534-0 (PMC9933400; doi:10.1186/s12915-023-01534-0)
Supplement: Supplementary file 13 — Additional file 13: Legends for Additional files 1–12. [file 12915_2023_1534_MOESM13_ESM.docx]

**Legends for Additional files 1–12**

**Additional file 1: Figure S1. Scm is required for dendrite pruning in ddaC neurons.**

(A) Lethal stages of *Scm* allelic combinations.

(B) Live confocal images of ddaC neurons at 18 h APF. Somas of ddaC are marked by red arrowheads. Overexpression of full-length Scm significantly rescued the pruning defects in *Scm^H3885^* homozygous mutant ddaC neurons. Quantification of severing and fragmentation defects, length of unpruned ddaC dendrites at 18 h APF.

(C) Live confocal images of ddaC neurons at 16 h APF. Somas of ddaCs are marked by red arrowheads. Knockdown of *Scm* by three independent RNAi lines caused dendrite pruning defects in ddaC neurons at 16 h APF. Quantification of severing and fragmentation defects, length of unpruned ddaC dendrites at 16 h APF.

(D) Confocal images of ddaC neurons (green) at wL3 stage immuno-stained with anti-Scm (magenta). RNAi knockdown of Scm (RNAi line #1) led to a significant reduction in its protein levels. Quantification of Scm protein levels in the nuclei of ddaC neurons.

The number of neurons (n) in each group is shown on the bars. Error bars in all experiments represent ±SEM. Two-tailed Student’s T-test was used to determine statistical significance for pairwise comparison, whereas one-way ANOVA with Bonferroni test was applied to determine significance for multiple-group comparison. ns, no significance, ***p<0.001. Three independent replicates were conducted. Scale bars represent 50 µm in (B-C) and 10 µm in (D).

**Additional file 2: Figure S2. *Scm* is required for dendrite pruning of class I ddaD/ddaE neurons but not for apoptosis of class III ddaF neurons.**

(A) Live confocal images of full-arbor morphology of control an*d Scm^D1^* ddaC MARCM clones at 96 h AEL. Quantification of dendrite termini numbers and sholl analysis.

(B) Live confocal images of ddaD MARCM clones labelled by mCD8GFP at WP stage and 20 h APF. ddaD somas are marked by red arrowheads. Control ddaD MARCM clones pruned all their dendrites at 20 h APF, whereas dendrites remained attached to the somas in *Scm^D1^* ddaD MARCM clones.

(C) Live confocal images of ddaF MARCM clones labelled by mCD8GFP at WP stage and 16 h APF. ddaF somas are marked by red arrowheads. Both control and *Scm^D1^* ddaF MARCM clones died at 16 h APF.

The number of neurons (n) in each group is shown on the bars. Error bars in all experiments represent ±SEM. Two-tailed Student’s T-test was used to determine statistical significance for pairwise comparison. *p<0.05. Three independent replicates were conducted. Scale bars represent 50 µm.

**Additional file 3: Figure S3. PRC1 is required for dendrite pruning in ddaC neurons.**

(A) Quantification of severing and fragmentation defects of *Psc*, *Sce,* *Su(z)2*, *ph-d* or *ph-p* RNAi expressing ddaC neurons at 16 h APF.

(B) Live confocal images of ddaC neurons at WP and 16 h APF. ddaC somas are marked by red arrowheads. Dendrites of control ddaC neurons were pruned away at 16 h APF, whereas two copies of *ph-d*, *ph* or *Pc* RNAi knockdown caused dendrite pruning defects in ddaC neurons. Quantification of severing and fragmentation defects, length of unpruned dendrites of ddaC neurons at 16 h APF.

(C) Live confocal images of full-arbor morphology of control an*d ph* RNAi expressing ddaC neurons at wL3. Quantification of dendrite termini numbers and sholl analysis.

(D) Live confocal images of ddaC neurons at 16 h APF. Somas of ddaCs are marked by red arrowheads. Dendrites of control ddaC neurons were pruned away at 16 h APF, whereas *Psc* and *Su(z)2* double RNAi knockdown caused dendrite pruning defects in ddaC neurons. Quantification of severing and fragmentation defects, length of unpruned dendrites of ddaC neurons at 16 h APF.

The number of samples (n) in each group is shown on the bars. Error bars in all experiments represent ±SEM. Two-tailed Student’s T-test was used to determine statistical significance for pairwise comparison, whereas one-way ANOVA with Bonferroni test was applied to determine significance for multiple-group comparison. **p<0.01, ***p<0.001. Three independent replicates were conducted. Scale bars represent 50 µm.

**Additional file 4: Figure S4. Ph silences Scr expression in ddaC neurons.**

(A) Confocal images of ddaC neurons (green) at eL3, wL3 and WP stages immuno-stained with anti-Ubx, Abd-A, anti-Abd-B or Scr (magenta). ddaC somas are marked by dashed lines and ddaE by asterisks. Ubx and Abd-A were expressed at lower levels in ddaC neurons at eL3, wL3 and WP stages, relative to those in other da neurons. Abd-B and Scr were fully repressed in ddaC and other da neurons.

(B) Confocal images of ddaC neurons (green) immuno-stained with anti-Abd-B or anti-Scr (magenta). ddaC somas are marked by dashed lines. Knockdown of *Abd-B* or *Scr* in *ph* RNAi expressing ddaC neurons showed significant reductions in Abd-B or Scr levels, respectively. Quantification of Abd-B and Scr protein levels in ddaC nuclei.

(C) MARCM ddaC clones (green) derived from *ph^505^* mutant allele immuno-stained with anti-Scr (magenta). Non-clonal heterozygous ddaC controls were taken from the contralateral segment of their respective clones. ddaC somas are marked by dashed lines. Quantification of Scr protein levels in the ddaC nuclei.

(D) Confocal images of ddaC neurons (green) immuno-stained with anti-Abd-B (magenta). ddaC somas are marked by dashed lines. *Psc* and *Su(z)2* double RNAi ddaC neurons showed de-repression of Abd-B. Quantification of Abd-B protein levels in ddaC nuclei.

The number of neurons (n) in each group is shown on the plots. Error bars in all experiments represent ±SEM. Two-tailed Student’s T-test was used to determine statistical significance for pairwise comparison. ***p<0.001. Three independent replicates were conducted. Scale bar represents 10 µm.

**Additional file 5: Figure S5. The PRC2 component E(z) is required for suppression of Ubx and Abd-A expression in ddaC neurons.**

(A-B) *E(z)^731^* MARCM ddaC clones (green) immuno-stained with anti-Ubx, anti-Abd-A*,* anti-Abd-B or anti-Scr (magenta). The ddaC somas are labelled by dashed lines and ddaE by asterisks. Non-clonal heterozygous ddaC controls were taken from the contralateral segment of their respective clones. Compared to the controls, Ubx and Abd-A levels, but not Abd-B and Scr, were upregulated in the *E(z)^731^* MARCM ddaC clones. Quantification of Ubx, Abd-A, Abd-B and Scr protein expression levels in the ddaC nuclei in the bottom charts.

The number of neurons (n) in each group is shown on the bars. Error bars in all experiments represent ±SEM. Two-tailed Student’s T-test was used to determine statistical significance for pairwise comparison. ns, no significance, **p<0.01, ***p<0.001. Three independent replicates were conducted. Scale bars represent 10 µm.

**Additional file 6: Figure S6. Scm is required for suppression of Ubx and Abd-A expression in ddaC neurons.**

(A-B) *Scm^D1^* MARCM ddaC clones (green) immuno-stained with anti-Ubx, anti-Abd-A*,* anti-Abd-B or anti-Scr (magenta). ddaC somas are labelled by dashed lines and ddaE by asterisks. Non-clonal heterozygous ddaC controls were taken from the contralateral segment of their respective clones. Compared to the controls, the expression levels of Ubx and Abd-A, but not Abd-B and Scr, were upregulated in the *Scm^D1^* MARCM ddaC clones. Quantification of Ubx, Abd-A, Abd-B and Scr protein expression levels in the ddaC nuclei in the bottom charts.

The number of neurons (n) in each group is shown on the bars. Error bars in all experiments represent ±SEM. Two-tailed Student’s T-test was used to determine statistical significance for pairwise comparison. ns, no significance, **p<0.01, ***p<0.001. Three independent replicates were conducted. Scale bars represent 10 µm.

**Additional file 7: Figure S7. Knockdown of Abd-B or Scr did not rescue the dendrite pruning defects in *ph* RNAi ddaC neurons.**

(A) Live confocal images of ddaC neurons at WP and 16 h APF. ddaC somas are indicated by red arrowheads. Knockdown of *Scr* or *Abd-B* did not suppress the dendrite pruning defects in *ph* RNAi ddaC neurons at 16 h APF. Quantification of severing and fragmentation defects, length of unpruned dendrites of ddaC neurons at 16 h APF.

(B) Confocal images of ddaC neurons (green) immune-stained with anti-Cut or anti-Knot (magenta). ddaC somas are marked by dashed lines. Cut and knot expression was downregulated in *ph* RNAi ddaC neurons at wL3. Quantification of Cut and Knot protein levels in ddaC nucleus.

(C) Confocal images of ddaC neurons (green) immuno-stained with anti-Mical (magenta). ddaC somas are labelled by dashed lines and ddaE by asterisks. Knockdown of *Abd-B* did not restore Mical expression in *ph* RNAi ddaC neurons at WP stage.

The number of neurons (n) in each group is shown on the bars. Error bars in all experiments represent ±SEM. Two-tailed Student’s T-test was used to determine statistical significance for pairwise comparison, whereas one-way ANOVA with Bonferroni test was applied to determine significance for multiple-group comparison. n.s., not significant, *p<0.05, ***p<0.001. Three independent replicates were conducted. Scale bars represent 50 µm in (A) and 10 µm in (B and C).

**Additional file 8: Figure S8. PRC1, but not PRC2, is important for Mical expression in ddaC neurons before pruning.**

(A) MARCM ddaC clones (green) derived from *Pc^15^*, *E(z)^731^, Su(z)12^2^* and *Scm^D1^* mutant alleles and ddaC neurons of *Psc* and *Su(z)2* double RNAi at WP stage immuno-stained anti-Mical (magenta). ddaC somas are marked by dashed lines. ddaE somas are marked with asterisks. Mical expression levels were unaffected in *E(z)^731^, Su(z)12^2^* and *Scm^D1^* MARCM ddaC clones but significantly reduced in *Pc^15^* MARCM ddaC clones and *Psc* and *Su(z)2* double RNAi ddaC neurons. Quantification of Mical expression levels.

(B) Expression of *mical1-lacZ* (magenta) in *ph* RNAi and Abd-B-overexpressing ddaC neurons at WP stage. LacZ expression was significantly reduced in *ph* RNAi and Abd-B-overexpressing ddaC neurons. Quantification of LacZ expression levels.

(C) Live confocal images of ddaC neurons at 20 h APF. ddaC somas are indicated by red arrowheads. Overexpression of *Mical^FL^* was able to partially suppress dendrite pruning defects of *ph* RNAi and Abd-B-overexpressing neurons. Quantification of severing and fragmentation defects, unpruned dendrite lengths at 20 h APF.

The number of neurons (n) in each group is shown on the bars. Error bars in all experiments represents ±SEM. Two-tailed Student’s T-test was used to determine statistical significance for pairwise comparison. n.s., not significant, *p<0.05, ***p<0.001. Three independent replicates were conducted. Scale bars represent 10 µm in (A and B) and 50 µm in (C).

**Additional file 9: Figure S9. Scm is required for axonal pruning in MB γ neurons.**

(A) Confocal images of MB γ neurons expressing mCD8GFP driven by *201Y-Gal4* and co-stained with anti-GFP (green) and anti-FasII (magenta) at wL3 stage and 24 h APF. White arrowheads point to the unpruned axons of γ neurons at 24 h APF as co-labelled by GFP and FasII. Axons of the control MB γ neurons were pruned away at 24 h APF, whereas *scm* RNAi MB γ neurons exhibited axon pruning defects.

(B) Confocal images of MB γ neurons expressing mCD8GFP co-stained with anti-GFP (green) and anti-Ubx, anti-Abd-A, anti-Abd-B or anti-Scr (magenta) at 6 h APF. Somas of MB γ neurons are labelled by dashed lines. Ubx, Abd-A, Abd-B and Scr were not expressed in either control or *ph* RNAi neurons.

Three independent replicates were conducted. Scale bars represent 10 µm.

**Additional file 10: Figure S10. A schematic representation summarizes the potential role of Ph and Abd-B proteins in regulating Mical expression and thereby ecdysone signalling during dendrite pruning.**

**Additional file 11: Table S1. Source data for all the figures.**

**Additional file 12: A list of fly strains used in all the figures.**
